# Clinical and Molecular Epidemiology of Extended-Spectrum Beta-Lactamase-Producing *Klebsiella* spp.: A Systematic Review and Meta-Analyses

**DOI:** 10.1371/journal.pone.0140754

**Published:** 2015-10-20

**Authors:** Tirza C. Hendrik, Anne F. Voor in ‘t holt, Margreet C. Vos

**Affiliations:** Department of Medical Microbiology and Infectious Diseases, Erasmus MC University Medical Center, Rotterdam, The Netherlands; California Department of Public Health, UNITED STATES

## Abstract

Healthcare-related infections caused by extended-spectrum beta-lactamase (ESBL)-producing *Klebsiella* spp. are of major concern. To control transmission, deep understanding of the transmission mechanisms is needed. This systematic review aimed to identify risk factors and sources, clonal relatedness using molecular techniques, and the most effective control strategies for ESBL-producing *Klebsiella* spp. A systematic search of PubMed, Embase, and Outbreak Database was performed. We identified 2771 articles from November 25th, 1960 until April 7th, 2014 of which 148 were included in the systematic review and 23 in a random-effects meta-analysis study. The random-effects meta-analyses showed that underlying disease or condition (odds ratio [OR] = 6.25; 95% confidence interval [CI] = 2.85 to 13.66) generated the highest pooled estimate. ESBL-producing *Klebsiella* spp. were spread through person-to-person contact and via sources in the environment; we identified both monoclonal and polyclonal presence. Multi-faceted interventions are needed to prevent transmission of ESBL-producing *Klebsiella* spp.

## Introduction

Healthcare-related infections (HRIs) are a major clinical problem worldwide. In 2011, the World Health Organization (WHO) reported that in a mixed patient population the pooled HRI-prevalence was 10.1% in low- and middle-income countries and 7.6% in high-income countries [1]. Prolonged hospital stay, higher costs, increased antimicrobial resistance, and risk of potentially life-threatening conditions indicate the enormous burden of HRIs [2]. Further, we are facing HRIs caused by multidrug-resistant gram-negative bacteria (MDR-GNB) without a parallel progression of the novel antibiotic classes [3].

*Klebsiella* spp. have been recognized as the most frequent cause of MDR-GNB outbreaks, particularly after the emergence of the extended-spectrum beta-lactamase (ESBL) enzymes [4, 5]. As a result, infections in hospitalized patients with this ESBL-producing *Klebsiella* spp. have raised public concern due to the clinical outcomes and limited antibiotic options [6]. Patients whose care requires devices, and patients who are identified with multiple antibiotic-resistant strains in the intensive care unit (ICU) are at highest risk to acquire an infection with an ESBL-producing *Klebsiella* spp. [7, 8]. High discriminatory subtyping methods are beneficial to determine clonality of the outbreak strains with pulsed-field gel electrophoresis (PFGE) as the well-known ‘gold standard’ for molecular epidemiological studies and for current clinical use [9].

It requires deep understanding of all outbreaks to optimally control transmission of ESBL-producing *Klebsiella* spp. [10]. Recent guidelines about the management of MDR-GNB underscore the need of well-managed and multi-faceted interventions [11]. Therefore, it is necessary to investigate the transmission dynamics and the risk factors for hospital outbreaks. This systematic review aimed to answer the following four questions. First, what are the risk factors for the presence of ESBL-producing *Klebsiella* spp.? Second, what are the main sources and reservoirs for this microorganism? Third, how can we identify the transmission patterns and the clonal relatedness among isolates from patients who acquired ESBL-producing *Klebsiella* spp.? Fourth, what are the most effective control strategies for ESBL-producing *Klebsiella* spp.?

## Materials and Methods

This systematic review and meta-analysis followed the guidelines outlined in the PRISMA statement (S1 File)[12].

### Search Strategy and Selection Criteria

We searched PubMed, Embase, and the Outbreak Database (until April 7^th^, 2014) to identify studies which examined the transmission of multidrug-resistant (MDR) *Klebsiella* spp., identified potential risk factors, described modes of transmission, described laboratory methods used for the identification, and described the effective interventions to prevent transmission of MDR *Klebsiella* spp. with using the terms as applied in S2 File. The search strategy was not limited by language, date of publication, country, study design, enzyme type, or patient characteristics. We excluded studies about: 1) pathogenesis, validation of molecular techniques, drug options, cost, 2) non-human studies, 3) studies only about carriers, health-care workers (HCWs), or family members, 4) studies only about environmental contamination, 5) case report with no statement on transmission, 6) non-hospital studies, 7) letters, editorials, communications, weekly reports, and reviews. However, we also searched the eligible citations of all relevant reviews. TCH initiated full searches and AFV independently repeated the search for a 5 percent subset of articles.

### Data Extraction

We first screened all articles based on titles and abstracts and then we subsequently assessed the articles in full text according to the inclusion and exclusion criteria. TCH initiated the screening and extracted the data with help of AFV and MCV. To retrieve articles that could not be found in full-text, we contacted first authors or corresponding authors of 80 publications. We also contacted the authors of 16 publications to obtain missing information about associated factors and cluster analyses. We defined the categories of MDR *Klebsiella* spp. as ESBL, possible ESBL and non-ESBL. We used the ESBL definition according to group 2b Bush criteria [13]. We found several articles that showed resistance to cephalosporins before the term ‘ESBL’ was established in 1989 [14]. These studies were included as being ‘ESBL’. Ultimately, we only focused on studies about ESBL-producing *Klebsiella* spp. within one hospital.

### Data Analyses

We included articles related to ESBL-producing *Klebsiella* spp. that described the factors associated with the presence or acquisition of ESBL-producing *Klebsiella* spp. using a multivariate model. We took into account studies that have suggested and proven the sources of ESBL-producing *Klebsiella* spp. using molecular typing techniques. However, we also included studies that suggested the potential reservoirs. In addition, we included studies about the associated factors for mortality related to ESBL-producing *Klebsiella* spp.

In order to assess clonal relatedness and transmission patterns of ESBL-producing *Klebsiella* spp., studies that only performed phenotypic typing methods were excluded and studies that did use molecular typing were included. We merged studies that used polymerase chain reaction (PCR)-based techniques for typing. We assessed the result of molecular typing methods and calculated the total number of identified patterns. We defined a cluster as ≥ two similar patterns of ESBL-producing *Klebsiella* spp. isolates. Likewise, a unique isolate was defined as a single pattern. The term monoclonal presence referred to a single cluster and the term polyclonal presence referred to ≥ two clusters. We calculated the total number of patterns, the clusters including cluster sizes, and the single patterns. If the information was available, we performed the cluster analyses based on the number of patients, otherwise on the number of isolates. We also reviewed studies about infection control strategies and prevention programs. We assessed the standard interventions possibly combined with additional control strategies, and reported which were most successful strategies to stop transmission. We compiled data from two studies that were presented in four publications in the result section [15–18].

### Statistical Analysis

We combined all associated factors that reported an odds ratio (OR) and a 95% confidence interval (95% CI) into ten different categories: 1, medical devices (e.g. mechanical ventilation, intra-vascular devices); 2, prior cephalosporin exposure; 3, prior quinolone exposure; 4, prior other antibiotic exposure; 5, prior antifungal exposure; 6, length of hospital stay; 7, patient characteristics (e.g. age); 8, underlying disease or condition (e.g. malignant disease); 9, medical procedures (e.g. surgical intervention); 10, other (e.g. exposure to the hands of HCWs). Studies reporting associated factors for mortality were excluded. Random-effects meta-analyses were performed for all categories except the “prior antifungal exposure”, “medical procedures” which had less than three factors and “other” that comprised many various factors. Lytsy et al., used three different models of multivariable analyses to find reliable estimates for the most important variables. However, we chose to only include model 1 in our meta-analyses [18]. We applied the method of DerSimonian and Laird and meta-analyses were performed using StatsDirect statistical software (StatsDirect, Version 2.8.0, Altrincham, StatsDirect Ltd, 2013) [19]. We considered *P* values <0.05 as statistically significant. Bias assessment plots were constructed to explore publication bias using the Egger and Begg-Mazumbdar (Kendall’s tau) indicators [19, 20].

### Study Quality

The methodological quality of all studies in the random-effects meta-analyses was assessed using the strengthening the reporting of observational studies in epidemiology (STROBE) guidelines (Table A and Table B in S3 File) or the Newcastle-Ottawa quality assessment scale (Table A and Table C in S3 File), based on the study design [21, 22]. Furthermore, the methodological quality of all molecular epidemiological studies in cluster analysis was assessed by the strengthening the reporting of molecular epidemiology for infectious disease (STROME-ID) guidelines (Table D in S3 File) [23]. However, the study quality was not considered as an exclusion criterion.

## Results

### Description

**Fig 1:**
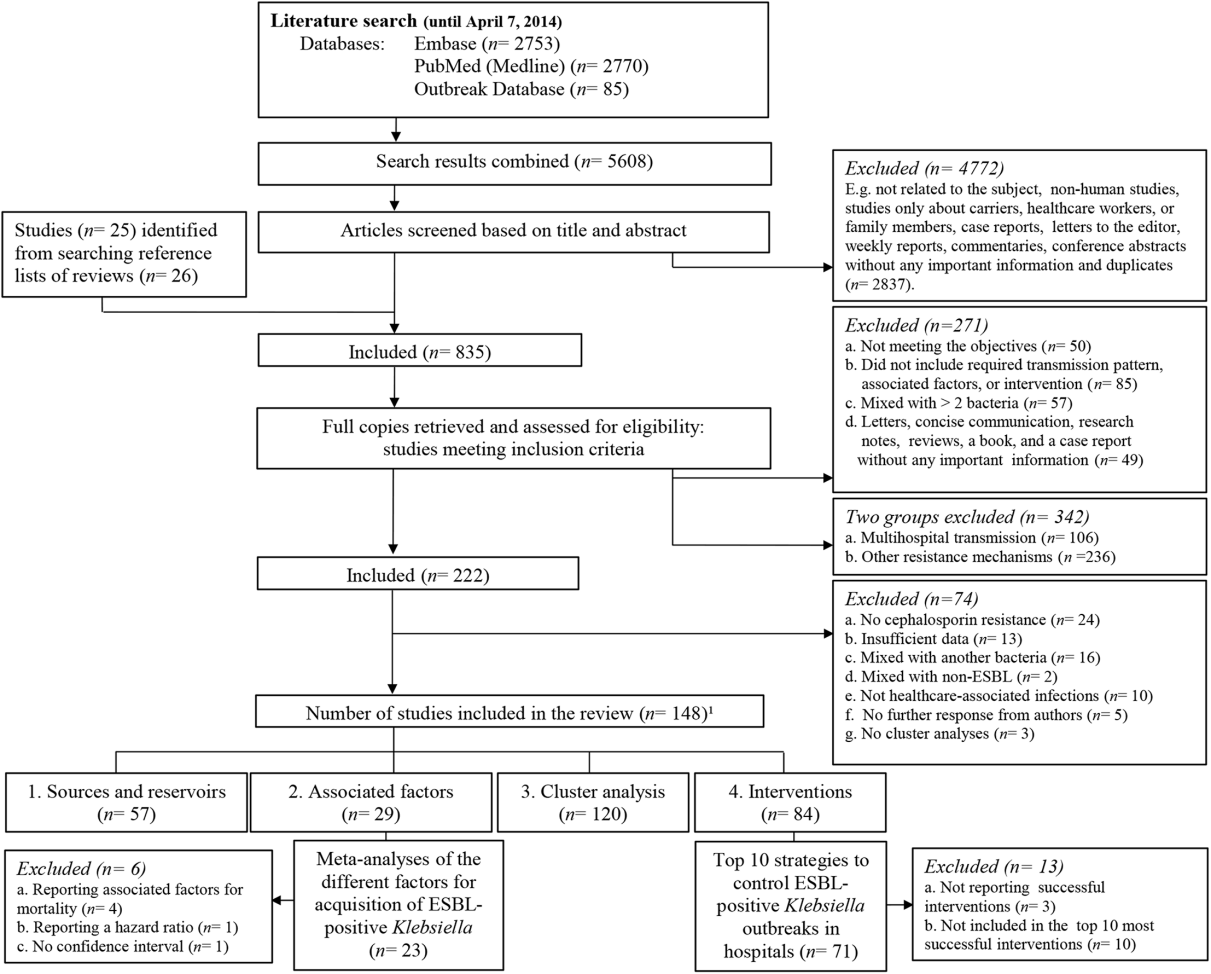
Flow diagram of study selection for the systematic review and random-effects meta-analyses on extended-spectrum beta-lactamase-producing *Klebsiella* spp. ^1^ Number of studies included in the review comprise the sources and reservoirs, associated factors, cluster analysis and successful interventions. ^2^ No response was obtained from the first and/or corresponding authors for the requested article.

We identified a total of 5,608 articles as potentially relevant when using our search strategy (Fig 1). Of these, 835 articles met the eligibility criteria and 25 articles were retrieved from citations of interesting reviews. We received 45 full-text articles from 80 authors. We got three further responses to the information requests that were sent to the authors of 17 articles. We ultimately included 148 articles in this systematic review (Fig 1). Five articles were written in Spanish, one article was written in French, one article was written in Turkish and 141 articles were written in English. The non-English articles were translated prior to data extraction. Most studies were conducted in Europe (39.9%; *n* = 59) particularly in France (*n* = 16), followed by Asia (18.9%; *n* = 28), North America (16.9%; *n* = 25), Africa (9.5%; *n* = 14), South America (10.8%; *n* = 16), multiple regions: Europe and Asia (3.4%; *n* = 5) and Australia (0.7%, *n* = 1) with 49 countries in total. All studies were published between 1991 and 2014 with study periods of less than a month up to seven years. Thirty-one studies (20.9%) indicated the suspected or identified index case. The study population was predominated by adult patients (45.3%, *n* = 67), followed by neonates (33.1%; *n* = 49), pediatric patients (5.4%; *n* = 8), and mix groups including the studies located in hospitals but which did not mention the study population (16.2%; *n* = 24). Seventy-six percent of all studies took place at the ICU (*n* = 113). These studies can be further divided in neonatal ICU (38.1%; *n* = 43), pediatric ICU (3.5%; *n* = 4), adult ICU (48.7%; *n* = 55) and mix ICU units (9.7%; *n* = 11). However, fifty-two studies were located both at ICU and non-ICU.

### Associated Factors

We identified 26 studies reporting associated risk factors with a statistically significant OR above one (Table 1) and seven studies reporting associated protective factors with a statistically significant OR below one (Table 2) for the presence of ESBL-producing *Klebsiella* spp. In addition, four studies identified the associated factors for bloodstream infections caused by ESBL-producing *K*. *pneumonia* (Table 1). In general, prior antibiotic exposure was the most common associated factor in all studies. Four studies reported associated factors for mortality [24–27]. It was published in these four studies that the presentation with septic shock had the highest odds ratio (205.99) [24].

**Table 1:** Associated risk factors for the presence of ESBL-producing *Klebsiella* spp. based on multivariate analyses.

Associated risk factor	No. of factors	RE	RE or RE range	No. of cases (range)	Studies
Underlying disease or condition	17	OR	1.04–60.60	26–292	^[^ ^42^ ^]; [^ ^43^ ^]; [^ ^44^ ^]; [^ ^45^ ^](2x); [^ ^18^ ^](6x); [^ ^46^ ^]; [^ ^47^ ^](4x); [^ ^48^ ^]^
Other antibiotic exposure	15	OR	1.55–95.21	10–292	^[^ ^49^ ^]; [^ ^24^ ^](^ ^a^ ^); [^ ^50^ ^]; [^ ^44^ ^]; [^ ^45^ ^]; [^ ^51^ ^](^ ^a^ ^); [^ ^52^ ^]; [^ ^34^ ^]; [^ ^53^ ^]; [^ ^54^ ^]^
	1	HR	4.60	206	^[^ ^55^ ^]^
Length of hospital stay	11	OR	1.05–12.60	18–80	^[^ ^35^ ^]; [^ ^43^ ^]; [^ ^51^ ^](^ ^a^ ^); [^ ^56^ ^]; [^ ^52^ ^]; [^ ^57^ ^]; [^ ^18^ ^](3x); [^ ^58^ ^](^ ^a^ ^)^
	1	HR	1.26	206	^[^ ^55^ ^]^
Medical devices	9	OR	2.11–5.23	18–292	^[^ ^59^ ^](2x); [^ ^24^ ^](^ ^a^ ^); [^ ^50^ ^]; [^ ^52^ ^]; [^ ^57^ ^]; [^ ^48^ ^](3x)^
Prior cephalosporin exposure	9	OR	4.51–7.60	17–88	^[^ ^42^ ^]; [^ ^50^ ^]; [^ ^60^ ^](^ ^a^ ^); [^ ^18^ ^](2x); [^ ^46^ ^](3x); [^ ^58^ ^](^ ^a^ ^)^
Other^b^	8	OR	1.66–9.30	18–94	^[^ ^61^ ^]; [^ ^35^ ^]; [^ ^45^ ^]; [^ ^52^ ^](2x); [^ ^28^ ^]; [^ ^46^ ^]; [^ ^47^ ^]^
Patient characteristics	3	OR	1.14–13.10	10–48	^[^ ^49^ ^]; [^ ^51^ ^](^ ^a^ ^); [^ ^16^ ^]^
	1	HR	1.57	206	^[^ ^55^ ^]^
Prior quinolone exposure	3	OR	2.86–25.37	30–78	^[^ ^50^ ^]; [^ ^54^ ^]; [^ ^58^ ^](^ ^a^ ^)^
Medical procedures^b^	2	OR	9.34–10.35	52–60	^[^ ^24^ ^](^ ^a^ ^); [^ ^53^ ^]^
Prior antifungal exposure^b^	2	OR	5.3–12	204	^[^ ^44^ ^](2x)^

Abbreviations: RE, risk estimate, ^2x^, ^3x^, ^4x^ or ^6x^, two, three, four or six different factors per reference.

^a^ Bloodstream infections.

^b^ This category was not included in a random-effects meta-analysis study.

**Table 2:** Associated protective factors for the presence of ESBL-producing *Klebsiella* spp. based on multivariate analyses.

Associated protective factor	No. of factors	No. of patients	RE	RE or RE Range	Studies
Medical devices	2	206	HR	0.22–0.52	^[^ ^55^ ^]^
Prior penicillin+ β lactamase inhibitor exposure^a^	2	88	OR	0.16–0.27	^[^ ^46^ ^]^
Others^b^	2	27–292	OR	0.22–0.50	^[^ ^34^ ^]; [^ ^48^ ^]^
Prior antibiotic exposure^a^	1	54	OR	0.003	^[^ ^56^ ^]^
Prior carbapenem exposure^a^	1	206	HR	0.22	^[^ ^55^ ^]^
Age^c^	1	47	OR	0.95	^[^ ^50^ ^]^
Prior cephalosporin exposure	1	204	OR	0.1	^[^ ^44^ ^]^

Abbreviations: RE, risk estimate; HR, hazard ratio; OR, odds ratio.

^a^ This factor was classified in the category of prior antibiotic exposure for a random-effects meta-analysis study.

^b^ This category was not included in a random-effects meta-analysis study.

^c^ This factor was classified in the category of patient characteristics for a random-effects meta-analysis study.

### Eight random-effects meta-analyses

**Fig 2:**
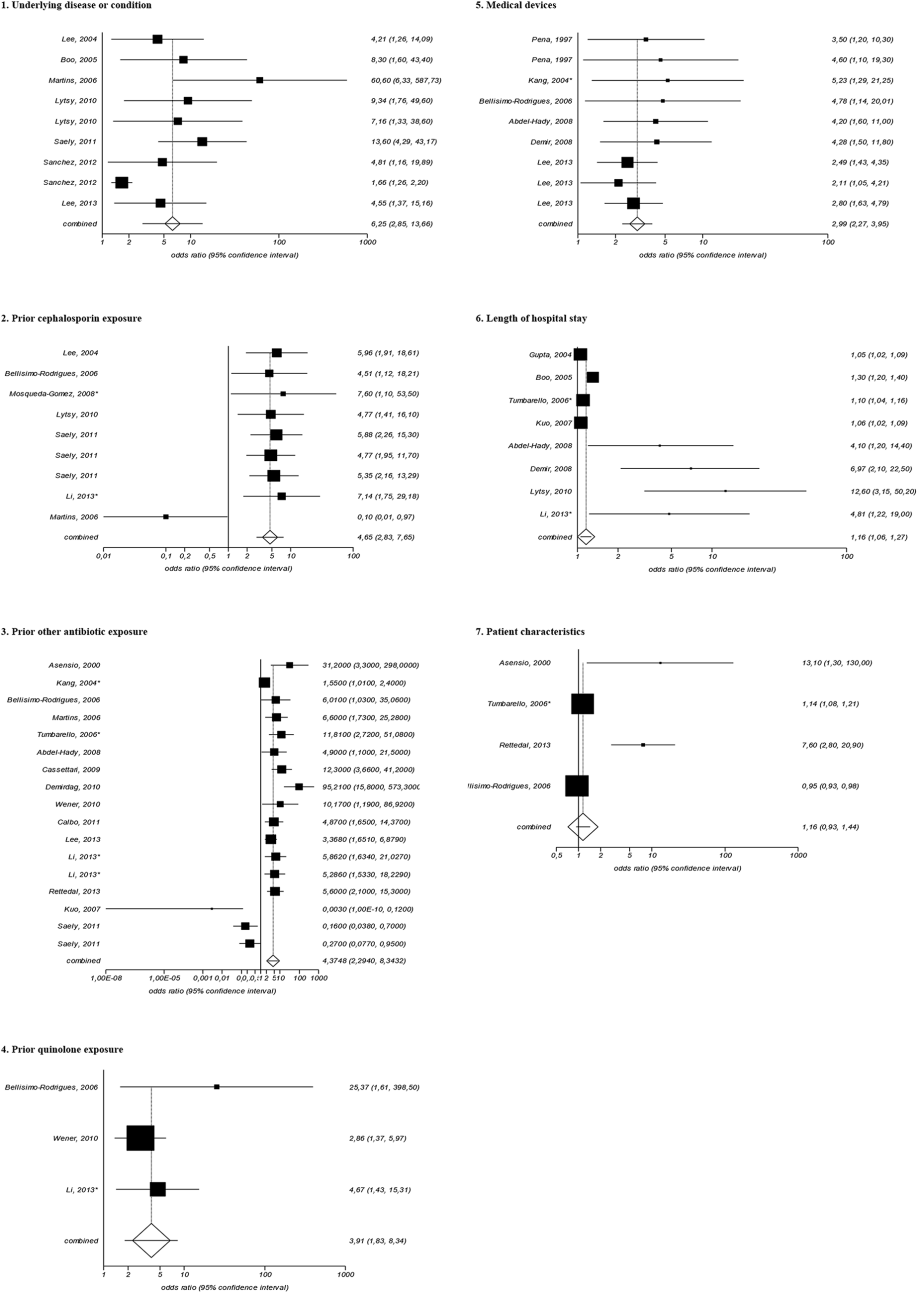
Forest plots of random-effects meta-analyses: individual and pooled odds ratios for associated risk factors and associated protective factors for presence of ESBL-producing Klebsiella spp. among patients in hospitals. * Bloodstream infections

Twenty-three studies were included in the seven random-effects meta-analyses, reporting 54 associated risk factors with a statistically significant OR above one and five associated protective factors with a statistically significant OR below one (Fig 1 and Table 3). The category of underlying disease or condition (OR = 6.25; 95% CI = 2.85 to 13.66) and prior cephalosporin exposure (OR = 4.65; 95% CI = 2.83 to 7.65) generated the highest pooled estimates (Fig 2). The publication bias indicators showed no significant results (Table 3).

**Table 3:** Random-effects meta-analyses of the different associated risk factors and associated protective factors for the presence of ESBL-producing *Klebsiella* spp. among patients in hospitals.

Associated factor	No. of factors^a^	Pooled OR	95% CI	Range of OR in individual studies	Risk of publication bias			
					Kendall's tau	P value	Egger	P value
Underlying disease or condition	9	6.25	2.85–13.66	1.66–60.60	0.39	0.18	2.61	< 0.01
Prior cephalosporin exposure	9	4.65	2.83–7.65	0.10–7.60	< 0.01	0.92	− 2.26	0.14
Prior other antibiotic exposure	17	4.38	2.29–8.34	0.003–95.21	0.16	0.39	1.51	0.16
Prior quinolone exposure	3	3.91	1.83–8.34	2.86–25.37	NC	NC	NC	NC
Medical devices	9	2.99	2.27–3.95	2.11–5.23	0.33	0.26	1.53	< 0.01
Length of hospital stay	8	1.16	1.06–1.27	1.05–12.60	0.55	0.08	3.25	< 0.01
Patient characteristics	4	1.16	0.93–1.44	0.95–13.10	< 0.01	0.75	4.11	0.18

Abbreviations: OR, odds ratio; CI, confidence interval; NC, not calculated because there were too few strata.

^a^ Only studies included that reported 95% CI and *P* values

### Sources and reservoirs

Fifty-seven studies identified environmental sources and/or surrounding reservoirs for ESBL-producing *Klebsiella* spp. (Table 4). Contaminated sinks were the most reported sources in the environment (13.8%; *n* = 4) whereas the patients were the main reservoirs (48.9%; *n* = 23), followed by the hands of HCWs (25.5%; *n* = 12). Interestingly, one study showed food as a transmission source for ESBL-producing *K*. *pneumonia* [28].

**Table 4:** Environmental sources and reservoirs for ESBL-producing *Klebsiella* spp., identified from *n* = 57 studies.

Reservoirs or Sources	No of studies	Studies
**Innate Environment**		
Bottles	1	^[^ ^62^ ^]^
Door handles, a siphon and a table	1	^[^ ^36^ ^](^ ^a^ ^)^
Entire unit (Intensive Care Unit)	2	^[^ ^63^ ^];[^ ^64^ ^]^
Hospital kitchen-screened surfaces	1	^[^ ^28^ ^](^ ^a^ ^)^
Incubator	2	^[^ ^65^ ^](^ ^a^ ^); [^ ^52^ ^](^ ^a^ ^)^
Liquid soap	2	^[^ ^66^ ^](^ ^a^ ^); [^ ^67^ ^](^ ^a^ ^)^
Mask	1	^[^ ^68^ ^](^ ^a^ ^)^
Moist surfaces near sinks and faucets	1	^[^ ^69^ ^](^ ^a^ ^)^
Roll boards in operating rooms	1	^[^ ^70^ ^](^ ^a^ ^)^
Room surface	2	^[^ ^65^ ^](^ ^a^ ^); [^ ^52^ ^](^ ^a^ ^)^
Single use products		
A. Intravenous medication or solution (re-used repeatedly)	2	^[^ ^71^ ^](^ ^a^ ^); [^ ^72^ ^](^ ^a^ ^)^
B. Intravenous glucose preparation (multiple-dosed)	1	^[^ ^62^ ^]^
C. Oxygen saturation probes (re-used repeatedly)	1	^[^ ^38^ ^](^ ^a^ ^)^
Sinks	4	^[^ ^73^ ^](^ ^a^ ^); [^ ^65^ ^](^ ^a^ ^); [^ ^74^ ^](^ ^a^ ^); [^ ^75^ ^](^ ^a^ ^)^
Suction pump located in the room of an infected patient	1	^[^ ^76^ ^](^ ^a^ ^)^
Suction tube	1	^[^ ^52^ ^](^ ^a^ ^)^
Thermometers	2	^[^ ^38^ ^](^ ^a^ ^); [^ ^77^ ^]^
Two water reservoirs from humidifiers	1	^[^ ^78^ ^](^ ^a^ ^)^
Ultrasonography coupling gel container of the emergency room	1	^[^ ^79^ ^](^ ^a^ ^)^
Weighing scale machine for babies	1	^[^ ^80^ ^](^ ^a^ ^)^
**Human**		
Transient Hand Carriage	1	^[^ ^81^ ^]^
A. Patients	23	^[^ ^82^ ^]; [^ ^83^ ^](^ ^a^ ^); [^ ^73^ ^](^ ^a^ ^); [^ ^84^ ^](^ ^a^ ^); [^ ^39^ ^](^ ^a^ ^); [^ ^61^ ^](^ ^a^ ^); [^ ^85^ ^](^ ^a^ ^); [^ ^86^ ^](^ ^a^ ^); [^ ^87^ ^](^ ^a^ ^); [^ ^88^ ^]; [^ ^49^ ^](^ ^a^ ^); [^ ^89^ ^](^ ^a^ ^);[^ ^90^ ^](^ ^a^ ^); [^ ^91^ ^](^ ^a^ ^); [^ ^92^ ^](^ ^a^ ^); [^ ^93^ ^](^ ^a^ ^); [^ ^94^ ^](^ ^a^ ^); [^ ^44^ ^](^ ^a^ ^); [^ ^95^ ^]; [^ ^96^ ^](^ ^a^ ^); [^ ^97^ ^](^ ^a^ ^); [^ ^98^ ^](^ ^a^ ^); [^ ^99^ ^](^ ^a^ ^)^
B. Health Care Workers	4	^[^ ^90^ ^](^ ^a^ ^); [^ ^100^ ^](^ ^a^ ^); [^ ^98^ ^](^ ^a^ ^); [^ ^101^ ^]^
Food handlers	1	^[^ ^28^ ^](^ ^a^ ^)^
Handholding due to work overcharge	1	^[^ ^102^ ^]^
Hands	11	^[^ ^103^ ^]; [^ ^90^ ^]; [^ ^77^ ^]; [^ ^65^ ^](^ ^a^ ^); [^ ^52^ ^](^ ^a^ ^); [^ ^57^ ^](^ ^a^ ^); [^ ^64^ ^]; [^ ^104^ ^](^ ^a^ ^); [^ ^76^ ^](^ ^a^ ^); [^ ^67^ ^](^ ^a^ ^); [^ ^105^ ^]^
Artificial nails	1	^[^ ^35^ ^](^ ^a^ ^)^
Onychomycosis	2	^[^ ^106^ ^](^ ^a^ ^); [^ ^107^ ^]&[^ ^34^ ^](^ ^a^ ^)^
C. Family (Mother to Child)		
Breast milk	2	^[^ ^38^ ^](^ ^a^ ^); [^ ^15^ ^](^ ^a^ ^)^
Peripartum colonization of neonates	1	^[^ ^79^ ^](^ ^a^ ^)^

^a^ This study proved the source or reservoir with use of a molecular typing technique.

### Cluster Analyses

One hundred-twenty studies described the molecular methods used to type ESBL-producing *Klebsiella* spp. and the analyses of genetic similarity (Table 5). In particular for ESBL-producing *K*. *pneumoniae*, ninety-two studies used PFGE and 16 studies used PCR-based techniques which were predominated by enterobacterial repetitive intergenic consensus sequence–polymerase chain reaction (ERIC-PCR) in seven studies. Multilocus sequence typing (MLST) was performed in seven studies as well as random amplified polymorphic DNA (RAPD). Nineteen studies performed more than one molecular method to analyze clusters. The average number of patients in the 120 studies was 22.5 (ranging from 1 to 295 patients). The median range of the number of patterns was 1 to 10 (median range of cluster-size medians from 1 to 15). In 111 studies that provided both number of clusters and the cluster size based on the number of patients, the median number of clusters was 2 ranging from 0 to 15 and the median of the cluster-size medians was 6 with a range of 0 to 81. In particular for *K*. *pneumoniae*, the median number of clusters was 2 and the median of cluster-size medians was 6. Further, the median number of clusters and the median of the cluster-size medians were 0 for *K*. *oxytoca*. Three surveillance studies reported an identical pattern indicating the clonality of identified ESBL-producing *Klebsiella* spp. strains in hospitals. In particular for the outbreak events, 43 studies showed monoclonal and 34 studies showed polyclonal presence of ESBL-producing *Klebsiella* spp. among patients within the hospital.

**Table 5:** Summary of studies (*n* = 120) reporting cluster analyses on identified ESBL-producing *Klebsiella* spp. using typing techniques.

Methods		No. of Strains:	No. of Patterns:	No. of Clusters:	Cluster Size:	Unique Isolates	No of Studies	Studies
		Median (Range)	Median (Range)^a^	Median (Range)	Median of Medians (Range of Median)	Median (Range)		
**Based on the number of patients**								
A. *K*. *pneumonia*								
PFGE		25 (2–235)	4 (1–55)	2 (0–15)	6 (0–81)	2 (0–45)	92	^[^ ^81^ ^](^ ^b^ ^,^ ^c^ ^); [^ ^108^ ^](^ ^b^ ^,^ ^c^ ^); [^ ^109^ ^]; [^ ^84^ ^](^ ^e^ ^); [^ ^39^ ^]; [^ ^79^ ^](^ ^b^ ^,^ ^c^ ^); [^ ^110^ ^](^ ^b^ ^,^ ^d^ ^,^ ^g^ ^); [^ ^61^ ^](^ ^b^ ^,^ ^c^ ^); [^ ^86^ ^](^ ^b^ ^,^ ^c^ ^); [^ ^87^ ^]; [^ ^88^ ^](^ ^b^ ^,^ ^c^ ^); [^ ^111^ ^](^ ^b^ ^,^ ^d^ ^,^ ^e^ ^); [^ ^37^ ^](^ ^b^ ^,^ ^d^ ^); [^ ^90^ ^](^ ^b^ ^,^ ^c^ ^); [^ ^112^ ^]; [^ ^66^ ^](^ ^b^ ^,^ ^c^ ^); [^ ^113^ ^](^ ^b^ ^,^ ^c^ ^,^ ^l^ ^,^ ^m^ ^); [^ ^114^ ^]; [^ ^89^ ^](^ ^b^ ^,^ ^c^ ^); [^ ^78^ ^](^ ^b^ ^,^ ^c^ ^); [^ ^115^ ^](^ ^b^ ^,^ ^c^ ^); [^ ^91^ ^](^ ^b^ ^,^ ^c^ ^); [^ ^116^ ^](^ ^b^ ^,^ ^d^ ^); [^ ^102^ ^](^ ^b^ ^,^ ^d^ ^); [^ ^117^ ^](^ ^b^ ^,^ ^c^ ^); [^ ^118^ ^](^ ^b^ ^,^ ^c^ ^,^ ^f^ ^); [^ ^119^ ^]; [^ ^55^ ^](^ ^b^ ^,^ ^d^ ^); [^ ^120^ ^]; [^ ^100^ ^](^ ^b^ ^,^ ^c^ ^); [^ ^121^ ^](^ ^b^ ^,^ ^d^ ^); [^ ^35^ ^](^ ^b^ ^,^ ^c^ ^); [^ ^122^ ^](^ ^g^ ^,^ ^k^ ^,^ ^l^ ^,^ ^m^ ^); [^ ^123^ ^]; [^ ^106^ ^](^ ^b^ ^,^ ^c^ ^); [^ ^77^ ^](^ ^b^ ^,^ ^d^ ^); [^ ^92^ ^](^ ^b^ ^,^ ^d^ ^); [^ ^93^ ^](^ ^b^ ^,^ ^d^ ^); [^ ^122^ ^](^ ^g^ ^,^ ^k^ ^,^ ^l^ ^,^ ^m^ ^); [^ ^123^ ^]; [^ ^106^ ^](^ ^b^ ^,^ ^c^ ^); [^ ^77^ ^](^ ^b^ ^,^ ^d^ ^); [^ ^92^ ^](^ ^b^ ^,^ ^d^ ^); [^ ^93^ ^](^ ^b^ ^,^ ^d^ ^); [^ ^25^ ^](^ ^m^ ^); [^ ^65^ ^](^ ^e^ ^); [^ ^80^ ^](^ ^b^ ^,^ ^c^ ^); [^ ^44^ ^]; [^ ^124^ ^](^ ^b^ ^,^ ^c^ ^); [^ ^125^ ^]; [^ ^126^ ^]; [^ ^127^ ^](^ ^g^ ^); [^ ^57^ ^]; [^ ^128^ ^]; [^ ^96^ ^](^ ^b^ ^,^ ^d^ ^); [^ ^17^ ^] and [^ ^18^ ^](^ ^b^ ^,^ ^c^ ^,^ ^e^ ^,^ ^i^ ^); [^ ^129^ ^](^ ^b^ ^,^ ^d^ ^); [^ ^60^ ^](^ ^g^ ^); [^ ^130^ ^](^ ^b^ ^,^ ^d^ ^); [^ ^131^ ^](^ ^b^ ^,^ ^c^ ^); [^ ^97^ ^](^ ^b^ ^,^ ^d^ ^,^ ^e^ ^,^ ^l^ ^,^ ^m^ ^); [^ ^132^ ^](^ ^b^ ^,^ ^d^ ^); [^ ^133^ ^](^ ^e^ ^); [^ ^27^ ^](^ ^e^ ^); [^ ^98^ ^](^ ^e^ ^,^ ^m^ ^); [^ ^134^ ^]; [^ ^104^ ^](^ ^b^ ^,^ ^c^ ^); [^ ^135^ ^]; [^ ^53^ ^](^ ^l^ ^,^ ^m^ ^); [^ ^136^ ^](^ ^b^ ^,^ ^d^ ^); [^ ^137^ ^]; [^ ^138^ ^]; [^ ^139^ ^](^ ^b^ ^,^ ^d^ ^); [^ ^76^ ^](^ ^b^ ^,^ ^d^ ^); [^ ^28^ ^](^ ^b^ ^,^ ^d^ ^); [^ ^140^ ^](^ ^g^ ^); [^ ^141^ ^]; [^ ^142^ ^](^ ^b^ ^,^ ^c^ ^); [^ ^72^ ^](^ ^b^ ^,^ ^c^ ^,^ ^m^ ^); [^ ^143^ ^](^ ^b^ ^,^ ^d^ ^); [^ ^144^ ^]; [^ ^145^ ^]; [^ ^15^ ^] and [^ ^16^ ^] (^ ^b^ ^,^ ^d^ ^,^ ^i^ ^); [^ ^47^ ^](^ ^b^ ^,^ ^c^ ^); [^ ^75^ ^](^ ^b^ ^,^ ^c^ ^); [^ ^146^ ^](^ ^b^ ^,^ ^c^ ^); [^ ^147^ ^]; [^ ^148^ ^](^ ^l^ ^); [^ ^149^ ^](^ ^b^ ^,^ ^c^ ^); [^ ^150^ ^](^ ^b^ ^,^ ^d^ ^); [^ ^151^ ^]; [^ ^152^ ^](^ ^b^ ^,^ ^c^ ^,^ ^e^ ^); [^ ^153^ ^](^ ^b^ ^,^ ^d^ ^,^ ^g^ ^,^ ^k^ ^,^ ^l^ ^,^ ^m^ ^)^
PCR		24 (4–295)	4 (1–125)	1 (0–10)	5.5 (1–87)	2.5 (0–21)	16	^[^ ^154^ ^](^ ^b^ ^,^ ^d^ ^,^ ^e^ ^); [^ ^39^ ^]; [^ ^155^ ^](^ ^b^ ^,^ ^c^ ^); [^ ^156^ ^](^ ^b^ ^,^ ^c^ ^); [^ ^117^ ^](^ ^2x^ ^,^ ^b^ ^,^ ^c^ ^,^ ^n^ ^); [^ ^157^ ^](^ ^b^ ^,^ ^c^ ^); [^ ^44^ ^]; [^ ^80^ ^](^ ^b^ ^,^ ^c^ ^); [^ ^158^ ^](^ ^b^ ^,^ ^c^ ^,^ ^g^ ^); [^ ^159^ ^](^ ^m^ ^); [^ ^160^ ^](^ ^b^ ^,^ ^d^ ^); [^ ^99^ ^](^ ^b^ ^,^ ^c^ ^); [^ ^101^ ^](^ ^e^ ^,^ ^g^ ^); [^ ^161^ ^](^ ^e^ ^); [^ ^162^ ^](^ ^g^ ^); [^ ^150^ ^](^ ^b^ ^,^ ^c^ ^)^
RAPD		18 (8–40)	3 (1–17)	1 (1–4)	11 (7–19)	1 (0–15)	7	^[^ ^85^ ^](^ ^b^ ^,^ ^c^ ^); [^ ^49^ ^](^ ^b^ ^,^ ^c^ ^); [^ ^38^ ^](^ ^b^ ^,^ ^c^ ^); [^ ^37^ ^](^ ^b^ ^,^ ^d^ ^); [^ ^115^ ^](^ ^b^ ^,^ ^c^ ^); [^ ^100^ ^](^ ^b^ ^,^ ^c^ ^); [^ ^163^ ^](^ ^b^ ^,^ ^d^ ^)^
MLST		21.5 (1–46)	2.5 (1–15)	2 (0–3)	2 (0–19)	1 (0–13)	7	^[^ ^164^ ^]; [^ ^134^ ^]; [^ ^136^ ^](^ ^h^ ^); [^ ^15^ ^] and [^ ^16^ ^](^ ^b^ ^,^ ^d^ ^,^ ^i^ ^); [^ ^162^ ^](^ ^g^ ^); [^ ^150^ ^](^ ^b^ ^,^ ^d^ ^,^ ^l^ ^); [^ ^165^ ^](^ ^b^ ^,^ ^f^ ^)^
Ribotyping		18 (8–57)	5.5 (1–15)	2 (1–6)	6 (2–14)	2.5 (0–9)	6	^[^ ^103^ ^](^ ^b^ ^,^ ^d^ ^); [^ ^73^ ^](^ ^b^ ^,^ ^c^ ^); [^ ^166^ ^](^ ^b^ ^,^ ^d^ ^); [^ ^154^ ^](^ ^b^ ^,^ ^d^ ^,^ ^e^ ^); [^ ^77^ ^](^ ^b^ ^,^ ^d^ ^); [^ ^128^ ^]^
ME-AFLP		8 (-)	1 (-)	1 (-)	8 (-)	0 (-)	1	^[^ ^70^ ^](^ ^b^ ^,^ ^c^ ^)^
MLEE		19 (-)	11 (-)	1 (-)	9 (-)	10 (-)	1	^[^ ^167^ ^](^ ^b^ ^,^ ^c^ ^)^
B. *K*. *Oxytoca*								
PFGE		2 (1–101)	1 (1–27)	1 (0–3)	1 (0–8)	1 (0–24)	7	^[^ ^37^ ^](^ ^b^ ^,^ ^c^ ^); [^ ^122^ ^](^ ^g^ ^); [^ ^125^ ^]; [^ ^126^ ^]; [^ ^129^ ^](^ ^b^ ^,^ ^c^ ^); [^ ^139^ ^](^ ^b^ ^); [^ ^74^ ^](^ ^b^ ^,^ ^d^ ^,^ ^l^ ^)^
RAPD		2 (-)	1 (-)	1 (-)	2 (-)	0 (-)	1	^[^ ^37^ ^](^ ^b^ ^,^ ^c^ ^)^
**Based on the number of clinical strains**								
A. *K*. *pneumonia*								
PFGE		24.5 (18–31)	2 (2–2)	2 (1–2)	11 (9–13)	0 (0)	3	^[^ ^168^ ^](^ ^b^ ^,^ ^c^ ^,^ ^m^ ^); [^ ^165^ ^](^ ^b^ ^,^ ^f^ ^); [^ ^169^ ^](^ ^b^ ^,^ ^d^ ^,^ ^f^ ^)^
RAPD		37 (-)	28 (-)	6 (-)	2 (-)	22 (-)	1	^[^ ^94^ ^](^ ^f^ ^)^
B. *K*. *oxytoca*								
PFGE		13 (3–23)	5 (1–9)	1 (0–2)	3.5 (0–7)	1 (1–1)	2	^[^ ^69^ ^](^ ^f^ ^); [^ ^165^ ^](^ ^b^ ^,^ ^f^ ^)^
**Based on the number of patients or clinical strains + family and/or environmental strains**								
*K*. *pneumonia*								
PFGE		49 (30–49)	10 (2–25)	7 (2–7)	7 (2–15)	3 (0–18)	3	^[^ ^163^ ^](^ ^b^ ^,^ ^d^ ^); [^ ^170^ ^](^ ^b^ ^,^ ^d^ ^,^ ^e^ ^); [^ ^36^ ^](^ ^b^ ^,^ ^d^ ^,^ ^f^ ^)^
PCR		39 (30–48)	9.5 (2–17)	3 (2–4)	15 (-)	6.5 (0–13)	2	^[^ ^51^ ^](^ ^j^ ^,^ ^l^ ^); [^ ^36^ ^](^ ^b^ ^,^ ^d^ ^,^ ^f^ ^)^
Raman spectroscopy		30 (-)	2 (-)	2 (-)	15 (-)	0 (-)	1	^[^ ^36^ ^](^ ^b^ ^,^ ^d^ ^,^ ^f^ ^)^

Abbreviations: PFGE, pulsed-field gel electrophoresis; PCR, polymerase chain reaction (ERIC-PCR, enterobacterial repetitive intergenic consensus sequence; AP-PCR, arbitrarily primed-PCR; REP-PCR, repetitive sequence-based PCR; BOX-PCR; IRS-PCR, infrequent-restriction-site PCR; RFLP-PCR, restriction fragment length polymorphism); RAPD, random amplified polymorphic DNA; MLST, multi-locus sequence typing; ME-AFLP, multi-enzyme amplified fragment length polymorphism; MLEE, multilocus enzyme electrophoresis; (-), Only 1 study was reported

^2x^, This study performed two rounds of ERIC-PCR on a different dataset.

^a^ We assessed the result of molecular typing methods and reported the total number of identified patterns as number of patterns

^b^ Outbreak

^c^ Monoclonal

^d^ Polyclonal

^e^ The number of patients were not literally written

^f^ Number of clinical isolates for cluster analysis due to available data or different time points of the isolates

^g^ Different presentation of data between text and figures on papers, hence we used data from the figures (except Liu 1998, Lavigne 2004, Wu 2006 and Cassettari 2009)

^h^ MLST was only performed on the index patient

^i^ Two publications used same isolates, hence, we combined the data

^j^ This study was performed on 48 patient samples that consisted of 46 healthcare-related infections and 2 unidentified samples

^k^ Data of number of patterns was not available

^l^ Data of cluster size was not available

^m^ Data of unique isolates was not available

^n^ Data of unique isolates on the second round was not available.

### Effective Interventions

The identification of ESBL-producing *Klebsiella* spp. transmission in hospitals should be followed by infection control strategies. However, not all studies provided detailed information regarding the interventions. We identified 84 studies that described the interventions during the study period. Twenty-eight studies reported the standard interventions that did not succeed. All but three studies described the additional and/or successful strategies to prevent the spread of ESBL-producing *Klebsiella* spp. in hospitals. Ultimately, we presented the top ten strategies to control ESBL-producing *Klebsiella* spp. in hospitals from 71 studies (Table 6). Reinforcement of hand hygiene (46.5%) was the most successful intervention in all studies, followed by adequate compliance with the antibiotic control programs (33.8%). Removal of contaminated tools was also found in the list of top 10 strategies to control ESBL-producing *Klebsiella* spp. infections within the hospital.

**Table 6:** Top 10 strategies to control ESBL-producing *Klebsiella* spp. outbreaks in hospitals.

No.	Intervention	No. of studies	Studies
1	Reinforcement of hand hygiene	33	^[^ ^103^ ^]; [^ ^171^ ^]; [^ ^81^ ^]; [^ ^73^ ^]; [^ ^85^ ^]; [^ ^86^ ^]; [^ ^172^ ^]; [^ ^88^ ^]; [^ ^155^ ^]; [^ ^166^ ^]; [^ ^49^ ^]; [^ ^90^ ^]; [^ ^66^ ^]; [^ ^115^ ^]; [^ ^116^ ^]; [^ ^168^ ^]; [^ ^102^ ^]; [^ ^35^ ^](^ ^a^ ^); [^ ^77^ ^]; [^ ^92^ ^]; [^ ^157^ ^]; [^ ^62^ ^](^ ^a^ ^); [^ ^65^ ^]; [^ ^94^ ^](^ ^a^ ^); [^ ^80^ ^]; [^ ^44^ ^]; [^ ^95^ ^]; [^ ^64^ ^](^ ^a^ ^); [^ ^76^ ^];[^ ^67^ ^](^ ^a^ ^); [^ ^15^ ^]; [^ ^149^ ^]; [^ ^152^ ^]^
2	Control of antibiotic use	24	^[^ ^171^ ^]; [^ ^61^ ^]; [^ ^86^ ^]; [^ ^155^ ^]; [^ ^49^ ^]; [^ ^66^ ^]; [^ ^116^ ^]; [^ ^168^ ^]; [^ ^102^ ^]; [^ ^42^ ^]; [^ ^65^ ^]; [^ ^80^ ^]; [^ ^44^ ^]; [^ ^51^ ^]; [^ ^95^ ^]; [^ ^57^ ^]; [^ ^96^ ^](^ ^a^ ^); [^ ^76^ ^]; [^ ^67^ ^](^ ^a^ ^); [^ ^99^ ^]; [^ ^101^ ^]; [^ ^165^ ^]; [^ ^169^ ^]; [^ ^152^ ^]^
3	Strict hygienic practices	18	^[^ ^73^ ^]; [^ ^86^ ^]; [^ ^90^ ^]; [^ ^102^ ^]; [^ ^69^ ^](^ ^a^ ^); [^ ^92^ ^]; [^ ^173^ ^]; [^ ^94^ ^](^ ^a^ ^); [^ ^96^ ^](^ ^a^ ^); [^ ^131^ ^]; [^ ^76^ ^]; [^ ^141^ ^]; [^ ^15^ ^]; [^ ^47^ ^](^ ^a^ ^); [^ ^148^ ^](^ ^a^ ^); [^ ^149^ ^](^ ^a^ ^); [^ ^16^ ^]; [^ ^152^ ^]^
4	a. Screening programs with an active microbiological surveillance	15	^[^ ^49^ ^]; [^ ^90^ ^]; [^ ^38^ ^](^ ^a^ ^); [^ ^92^ ^]; [^ ^57^ ^]; [^ ^96^ ^](^ ^a^ ^); [^ ^131^ ^]; [^ ^139^ ^]; [^ ^76^ ^]; [^ ^99^ ^]; [^ ^15^ ^]; [^ ^47^ ^](^ ^a^ ^); [^ ^75^ ^](^ ^a^ ^); [^ ^148^ ^](^ ^a^ ^); [^ ^169^ ^]^
	b. Cohorting of patients	15	^[^ ^171^ ^]; [^ ^81^ ^]; [^ ^172^ ^]; [^ ^49^ ^]; [^ ^90^ ^]; [^ ^157^ ^](^ ^a^ ^); [^ ^65^ ^]; [^ ^95^ ^]; [^ ^96^ ^](^ ^a^ ^); [^ ^64^ ^](^ ^a^ ^); [^ ^131^ ^]; [^ ^15^ ^]; [^ ^148^ ^](^ ^a^ ^); [^ ^169^ ^]; [^ ^152^ ^]^
5	Single-use equipment	14	^[^ ^103^ ^]; [^ ^79^ ^](^ ^a^ ^); [^ ^88^ ^]; [^ ^155^ ^]; [^ ^166^ ^]; [^ ^49^ ^]; [^ ^116^ ^]; [^ ^77^ ^]; [^ ^92^ ^]; [^ ^71^ ^]; [^ ^76^ ^]; [^ ^141^ ^]; [^ ^72^ ^](^ ^a^ ^); [^ ^15^ ^]^
6	Barrier precautions	12	^[^ ^108^ ^]; [^ ^86^ ^]; [^ ^90^ ^]; [^ ^120^ ^]; [^ ^157^ ^](^ ^a^ ^); [^ ^70^ ^]; [^ ^80^ ^]; [^ ^44^ ^]; [^ ^76^ ^]; [^ ^141^ ^]; [^ ^47^ ^](^ ^a^ ^); [^ ^152^ ^]^
7	Patient isolation	11	^[^ ^73^ ^]; [^ ^85^ ^]; [^ ^155^ ^]; [^ ^49^ ^]; [^ ^90^ ^]; [^ ^102^ ^]; [^ ^95^ ^]; [^ ^76^ ^]; [^ ^36^ ^](^ ^a^ ^); [^ ^15^ ^]; [^ ^149^ ^](^ ^a^ ^)^
8	Good cooperation between department and infection control team	9	^[^ ^49^ ^]; [^ ^71^ ^]; [^ ^65^ ^]; [^ ^95^ ^]; [^ ^96^ ^](^ ^a^ ^); [^ ^131^ ^]; [^ ^142^ ^]; [^ ^149^ ^](^ ^a^ ^); [^ ^152^ ^]^
9	Personnel educational programs	8	^[^ ^88^ ^]; [^ ^90^ ^]; [^ ^66^ ^]; [^ ^71^ ^]; [^ ^80^ ^]; [^ ^95^ ^]; [^ ^76^ ^]; [^ ^169^ ^]^
10	a. Removal of contaminated tools	7	^[^ ^73^ ^]; [^ ^79^ ^](^ ^a^ ^); [^ ^69^ ^](^ ^a^ ^); [^ ^70^ ^]; [^ ^97^ ^](^ ^a^ ^); [^ ^15^ ^]; [^ ^75^ ^](^ ^a^ ^)^
	b. Temporary closure of contaminated rooms	7	^[^ ^174^ ^](^ ^a^ ^); [^ ^66^ ^]; [^ ^38^ ^](^ ^a^ ^); [^ ^92^ ^]; [^ ^94^ ^](^ ^a^ ^); [^ ^95^ ^]; [^ ^16^ ^]^
	c. Medical-equipment disinfections	7	^[^ ^90^ ^]; [^ ^78^ ^]; [^ ^44^ ^]; [^ ^175^ ^]; [^ ^141^ ^]; [^ ^72^ ^](^ ^a^ ^); [^ ^15^ ^]^

^a^ The study added this additional intervention that was successful to control the events of ESBL-producing *Klebsiella* spp. in the hospital.

## Discussion

### Summary of evidence

This is the first systematic review and meta-analyses to identify the transmission pattern of ESBL-producing *Klebsiella* spp. among hospitalized patients worldwide. Our random-effects meta-analyses showed the underlying disease including malignancy or particular condition (OR = 6.25; 95% CI = 2.85 to 13.66) and prior cephalosporin exposure (OR = 4.65; 95% CI = 2.83 to 7.65) as the most significant associated factors for the presence of ESBL-producing *Klebsiella* spp. Consistent with our study, Cornejo-Juárez et al., indicated the increased risk of ESBL-*Escherichia coli* bacteremia in patients with hematologic malignancies who had prior cephalosporins exposure [29]. Our finding was also in line with a systematic review of ESBL-producing *Enterobacteriaceae* in Latin America that reported prior antibiotic exposure, in particular cephalosporins as the associated risk factor with a statistically significant OR above one for the acquisition of ESBL-producing *Klebsiella* spp. [30]. Nevertheless, based on the study by Piroth et al., antibiotic use can be an associated protective factor with a statistically significant OR below one [31]. Moreover, our study reported the fact that infection with ESBL-producing *Klebsiella* spp. can cause fatal outcomes in association with other associated risk factors [32]. Our study confirmed the importance of interaction between hospitalized patients, environmental sources, and surrounding reservoirs, in particular the HCWs. Obviously, the colonized and/or infected patients were the consistent reservoirs (Table 1). Further, it was clear that surrounding reservoirs and sources must be taken into consideration in order to end the vicious cycle of the spread of ESBL-producing *Klebsiella* spp. In general, likewise in case of Methicillin-resistant *Staphylococcus aureus*, HCWs who are colonized with ESBL-producing *Klebsiella* spp. play a pivotal role in the transmission through their hands [33]. Even more, using artificial nails and having onychomycosis can be the transmission source for the presence of ESBL-producing *Klebsiella* spp. [34, 35].

#### Molecular typing

PFGE still is the gold standard for molecular typing, in particular to prove the transmission of ESBL-producing *Klebsiella* spp. among patients [9]. However, it is useful to perform another molecular technique in order to confirm the result of the main diagnostic technique or transmission [36, 37]. Furthermore, our findings showed that both monoclonal and polyclonal presence occur in outbreak events. This indicated that clonal dissemination plays a role in outbreaks [38]. However, it is also important to consider the horizontal plasmid transfer among different bacterial species [36]. Studies on plasmid fingerprinting showed the importance of plasmid genotyping to predict the mode of plasmid transmission among patients [37, 39].

#### Interventions

To stop the spread of ESBL-producing *Klebsiella* spp., reinforcement of hand hygiene and an antibiotic control program were the most successful interventions. Firstly, hand hygiene is a simple and low cost intervention to prevent the presence of ESBL-producing *Klebsiella* spp. The introduction of hand antiseptic will reduce the contamination of the hands with of ESBL-producing *Klebsiella* spp. [40]. Recent guidelines also highlight hand hygiene as the top priority to prevent the transmission of nosocomial infections [11]. Secondly, a meta-analysis study suggested cycling empirical antibiotic therapy to prevent antibiotic resistance [41]. This adjustable cycling model confirmed the antibiotic control program as an important strategy for ESBL-producing *Klebsiella* spp. transmission.

#### Study quality

Most studies included in the meta-analyses and cluster analysis had a low study quality using the recommended guidelines. However, in particular for the studies that were assessed with STROME-ID guidelines, they had wide range of key objectives that did not focus solely on molecular epidemiology of ESBL-producing *Klebsiella* spp. Nonetheless, reporting the cases of ESBL-producing *Klebsiella* spp. according to STROME-ID guidelines can give clear information about the evidence to detect the transmission dynamics with molecular typing and helps in making the health-policy decision. In general, the studies that were included answered our research questions; however, our study indicated the importance to report structured articles that follow appropriate guidelines for a high study quality.

### Strengths and Limitations

The major strength of our study was the inclusion of a large number of hospitalized patients with ESBL-producing *Klebsiella* spp. In the meta-analyses, we identified several risk factors that were associated with the presence of ESBL-producing *Klebsiella* spp. We also summarized the most successful interventions to prevent the spread of ESBL-producing *Klebsiella* spp., which to our knowledge has never been done before**.** However, our study also has some limitations. Firstly, the heterogeneity of all included studies in combination with the diverse models used in all individual statistical analyses, here combined in the meta-analyses. Therefore, we performed a random-effects model for the meta-analyses. Secondly, due to the different definitions of colonization and infection in the studies, we termed them as presence of ESBL-producing *Klebsiella* spp. As a consequence, reporting them separately was not possible. Thirdly, our study focused on clonal spread; hence, we did not include studies about plasmid transfers. Fourthly, some studies only performed molecular typing investigation on selected samples due to cost effectiveness. Therefore, most probably we have bias on the number of clusters. Fifthly, publication bias might have occurred since we have a broad range of articles from different objectives to comprehensively answer our research questions.

## Conclusion

The presence of ESBL-producing *Klebsiella* spp., which results in increased morbidity and mortality, may occur by either direct spread from patient to patient or indirect transmission via surrounding reservoirs and sources in the environment. Obviously, molecular typing techniques can identify transmission of ESBL-producing *Klebsiella* spp. within the hospital. Prior antibiotic exposure holds a key role for the presence of ESBL-producing *Klebsiella* spp. particularly cephalosporin use. Multi-faceted interventions, including reinforcement of hand hygiene and control of antibiotic use, are necessary to prevent the spread of ESBL-producing *Klebsiella* spp. Further studies on plasmid transfer are needed to learn more about transmission of ESBL-producing *Klebsiella* spp. within a hospital. In addition, it is important to report studies in a more structured way that systematically follows suitable guidelines.

## Supporting Information

S1 File PRISMA 2009 Checklist. (DOCX)

S2 File List of search terms. (DOCX)

S3 File Study quality. Quality assessment scores of the 23 articles that were included in the random-effects meta-analysis study (Table A). Quality assessment scores of the 23 articles that were included in the random-effects meta-analysis study, using the STROBE guidelines (Table B). Quality assessment scores of the 15 case-control studies that were included in the random-effects meta-analysis study, using the Newcastle Ottawa Scale (Table C1). Quality assessment scores of the three cohort studies that were included in the random-effects meta-analysis study, using the Newcastle Ottawa Scale (Table C2). Quality assessment scores of 120 articles that were included in the cluster analysis study (Table D).
(DOCX)
