# Supplementary material for: Clinical and Molecular Epidemiology of Extended-Spectrum Beta-Lactamase-Producing Klebsiella spp.: A Systematic Review and Meta-Analyses
Source: PLoS One. 2015 Oct 20;10(10):e0140754. doi: 10.1371/journal.pone.0140754 (PMC4617432; doi:10.1371/journal.pone.0140754)
Supplement: S2 File — (DOCX) [file pone.0140754.s002.docx]

**S2 File: List of search terms**

**PubMed**

Klebsiella AND (ESBL OR ESBL-positive OR ESBL-producing OR KPC OR Klebsiella pneumoniae carbapenemase OR IMP OR imipenemase OR VIM OR Verona integron–encoded metallo-β-lactamase OR OXA-48 OR NDM OR New Delhi metallo-beta-lactamase OR TEM OR SHV OR CTX-M OR extended-spectrum beta-lactamase OR extended spectrum beta lactamase OR extended spectrum beta lactamases OR antibiotic resistance OR resistance OR drug resistance OR multidrug resistance OR cephalosporin OR cefotaxime OR cefotaxim OR ceftazidime OR cefoperazone OR cefixime OR cefpodoxime OR ceftriaxone OR ceftriaxon OR monobactam OR carbapenem OR imipenem OR meropenem OR ertapenem OR multidrug-resistant OR drug resistant OR antimicrobial resistance) AND (transmission OR hospital transmission OR mode of transmission OR hospital associated infection OR hospital associated infections OR healthcare associated infection OR healthcare associated infections OR hospital acquired OR healthcare acquired OR healthcare related OR health care related infection OR hospital transmission dynamics OR hospital transmission dynamic OR nosocomial OR nosocomial infection OR nosocomial infections OR nosocomial transmission OR community OR secondary transmission OR contamination OR outbreak OR outbreaks OR hospital outbreak OR hospital outbreaks OR spread OR source OR carrier OR carriers OR epidemic) AND (virulence factor OR virulence factors OR pathogenicity factor OR pathogenicity factors OR risk factor OR risk factors OR molecular epidemiology OR epidemiology typing OR surveillance OR surveillance system OR case-control OR case control OR cohort OR descriptive study OR observational study OR observational OR epidemiology)

**EMBASE**

#1 ‘klebsiella’/exp OR klebsiella

#2 esbl OR ‘esbl-positive’ OR ‘esbl-producing’ OR kpc OR ‘klebsiella pneumoniae carbapenemase’ OR imp OR imipenemase OR vim OR ‘verona integron-encoded metallo-β-lactamase’ OR ‘oxa 48’ OR ndm OR ‘new delhi metallo-beta-lactamase’ OR tem OR shv OR ‘ctx-m’ OR ‘extended-spectrum beta-lactamase’ OR ‘extended spectrum beta lactamase’ OR ‘extended spectrum beta lactamases’ OR ‘antibiotic resistance’ OR resistance OR ‘drug resistance’ OR ‘multidrug resistance’ OR cephalosporin OR cefotaxime OR cefotaxim OR ceftazidime OR cefoperazone OR cefixime OR cefpodoxime OR ceftriaxone OR ceftriaxon OR monobactam OR carbapenem OR imipenem OR meropenem OR ertapenem OR ‘multidrug-resistant’ OR ‘drug resistant’ OR ‘antimicrobial resistance’

#3 transmission OR ‘hospital transmission’ OR ‘mode of transmission’ OR ‘hospital associated infection’ OR ‘hospital associated infections’ OR ‘healthcare associated infection’ OR ‘healthcare associated infections’ OR ‘hospital acquired’ OR ‘healthcare acquired’ OR ‘healthcare related’ OR ‘health care related infection’ OR ‘hospital transmission dynamics’ OR ‘hospital transmission dynamic’ OR nosocomial OR ‘nosocomial infection’ OR ‘nosocomial infections’ OR ‘nosocomial transmission’ OR community OR ‘secondary transmission’ OR contamination OR outbreak OR outbreaks OR ‘hospital outbreak’ OR ‘hospital outbreaks’ OR spread OR source OR carrier OR carriers OR epidemic

#4 ‘virulence factor’ OR ‘virulence factors’ OR ‘pathogenicity factor’ OR ‘pathogenicity factors’ OR ‘risk factor’ OR ‘risk factors’ OR ‘molecular epidemiology’ OR ‘epidemiology typing’ OR surveillance OR ‘surveillance system’ OR ‘case-control’ OR case control’ OR cohort OR ‘descriptive study’ OR ‘observational study’ OR observational OR epidemiology

Search: #1 AND #2 AND #3 AND #4
