# Supplementary material for: Clinical and Molecular Epidemiology of Extended-Spectrum Beta-Lactamase-Producing Klebsiella spp.: A Systematic Review and Meta-Analyses
Source: PLoS One. 2015 Oct 20;10(10):e0140754. doi: 10.1371/journal.pone.0140754 (PMC4617432; doi:10.1371/journal.pone.0140754)
Supplement: S3 File — Quality assessment scores of the 23 articles that were included in the random-effects meta-analysis study (Table A). Quality assessment scores of the 23 articles that were included in the random-effects meta-analysis study, using the STROBE guidelines (Table B). Quality assessment scores of the 15 case-control studies that were included in the random-effects meta-analysis study, using the Newcastle Ottawa Scale (Table C1). Quality assessment scores of the three cohort studies that were included in the random-effects meta-analysis study, using the Newcastle Ottawa Scale (Table C2). Quality assessment scores of 120 articles that were included in the cluster analysis study (Table D). (DOCX) [file pone.0140754.s003.docx]

**S3 File: Study Quality**

Quality assessment scores were performed for 23 studies in the random-effects meta-analyses. The Newcastle-Ottawa quality assessment scale for 15 case control studies showed that all resulted in score of 6 out of 10. In addition, one case control study had score of 5 out of 10. For cohort studies, two studies resulted in score of 5 and one study resulted in score of 7 out of a total of 13. The reasons for not getting a score were: 1) the use of hospital controls, 2) the use of medical records, 3) no information about non-respondents; 4) no information about follow-up patients. However, according to the STROBE guidelines, the median score for cohort studies (*n*=3) was 22 of a total 34 whereas the median score for case control studies (*n*=15) was 25 of a total 33. Further, the median score for cross-sectional studies (*n*=5) was 23 of a total 32. No information about potential confounders, effect modifiers, in addition to no further discussion about potential bias were the principal causes for lower scores.

Recent STROME-ID was applied to assess the study quality of 120 studies in the cluster analysis. When the information we gathered did not meet the full criteria of an STROME-ID item, the probability of scoring 1 on this item became 0.5. Two case reports had score of 6.5 and 10.5 out of 15 respectively. The mean score for 118 studies was 10.6 out of 19 (SD = 1.62). No study described any efforts to address discovery bias and any unique restrictions on the study sample size. Further, several reasons for not obtaining a score were: 1) the term use of molecular epidemiology; 2) no definition for key molecular terms; 3) no definition for the infectious-disease case; 4) no information about how the study dealt with missing data; 5) no dendogram or phylogenetic tree; 5) no report of the ethical consideration.

| **S3 Table A. Quality assessment scores of the 23 articles that were included in the random-effects meta-analysis study.** | | | |
| --- | --- | --- | --- |
| **Study^a^** | **Study Design** | **STROBE score: (n/max)^d^** | **Newcastle-Ottawa: (n/max)^e^** |
| Abdel-Hady (2008) | Cohort | 21/34 | 7/13 |
| Assensio (2000) | Case-control | 24/33 | 6/10 |
| Bellisimo-Rodrigues (2006) | Cross sectional | 25/32 | NA |
| Boo (2005) | Case-control | 25/33 | 5/10 |
| Calbo (2011) | Case-control | 24/33 | 6/10 |
| Cassettari (2009) | Cross sectional | 24/32 | NA |
| Demir (2008) | Case-control | 26/33 | 6/10 |
| Demirdag (2010) | Case-control | 26/33 | 6/10 |
| Gupta (2004) | Case-control | 24/33 | 6/10 |
| Kang (2004)^c^ | Case-control | 24/33 | 6/10 |
| Kuo (2007) | Case-control | 25/33 | 6/10 |
| Lee (2004) | Case-control | 26/33 | 6/10 |
| Lee (2013) | Cross-sectional^b^ | 23/32 | NA |
| Li (2013)^c^ | Cross-sectional^b^ | 23/32 | NA |
| Lytsy (2010) | Case-control | 28/33 | 6/10 |
| Martins (2006) | Cohort | 24/34 | 5/13 |
| Mosqueda-Gomez (2008)^c^ | Case-control | 24/33 | 6/10 |
| Pena (1997) | Cross-sectional^b^ | 23/32 | NA |
| Rettedal (2013) | Cohort^b^ | 22/34 | 5/13 |
| Saely (2011) | Case-control | 25/33 | 6/10 |
| Sanchez (2012) | Case-control | 26/33 | 6/10 |
| Tumbarello (2006) | Case-case-control | 25/33 | 6/10 |
| Wener (2010) | Case-case-control | 26/33 | 6/10 |

| \| Abbreviations: NA, not available because the coding was not for cross-sectional study.  ^a^ Studies are reported by first author and year. \| \| --- \| \| ^b^ The study design was not described in the paper. \| \| ^c^ Bloodstream infections. \| \| ^d^ n, study score; maximum score: cohort = 34, case control = 33, cross-sectional = 32. \| \| ^e^ n, study score; maximum score: cohort = 13, case control = 10. \| \|  \| | | | | | | | | | | | | | | | | | | | | | | | | | | | | | | | | | | | | | | | | |  |  |  |  |  |  |  |  |
| --- | --- | --- | --- | --- | --- | --- | --- | --- | --- | --- | --- | --- | --- | --- | --- | --- | --- | --- | --- | --- | --- | --- | --- | --- | --- | --- | --- | --- | --- | --- | --- | --- | --- | --- | --- | --- | --- | --- | --- | --- | --- | --- | --- | --- | --- | --- | --- | --- | --- | --- | --- | --- | --- | --- |
| **S3 Table B. Quality assessment scores of the 23 articles that were included in the random-effects meta-analysis study, using the STROBE guidelines.** | | | | | | | | | | | | | | | | | | | | | | | | | | | | | | | | | | |  |  |  |  |  |  | |  |  |  |  |  |  |  |
| **Study** | **1** | | **2** | **3** | **4** | **5** | **6** | | **7** | **8** | **9** | **10** | **11** | **12** | | | | | **13** | | | **14** | | | **15** | **16** | | | **17** | **18** | **19** | **20** | **21** | **22** |  |  |  |  |  |  |  |  |  |  |  |  |  |  |
|  | **a** | **b** |  |  |  |  | **a** | **b** |  |  |  |  |  | **a** | **b** | **c** | **d** | **e** | **a** | **b** | **c** | **a** | **b** | **c** |  | **a** | **b** | **c** |  |  |  |  |  |  |  |  |  |  |  |  |  |  |  |  |  |  |  |  |
| Abdel-Hady (2008) | − | 1 | 1 | 1 | 1 | 1 | 1 | − | − | 1 | − | 1 | 1 | 1 | 1 | N/A | N/A | 1 | 1 | 1 | N/A | − | 1 | − | 1 | 1 | 1 | N/A | − | 1 | − | 1 | 1 | − |  |  |  |  |  |  |  |  |  |  |  |  |  |  |
| Assensio (2000) | − | 1 | 1 | 1 | 1 | 1 | 1 | 1 | − | 1 | − | 1 | 1 | 1 | 1 | N/A | 1 | 1 | 1 | 1 | N/A | − | 1 |  | 1 | 1 | 1 | N/A | 1 | 1 | − | 1 | 1 | − |  |  |  |  |  |  |  |  |  |  |  |  |  |  |
| Bellisimo-Rodrigues (2006) | − | 1 | 1 | 1 | 1 | 1 | 1 |  | − | 1 | − | 1 | 1 | 1 | 1 | 1 | 1 | 1 | 1 | 1 | N/A | − | 1 |  | 1 | 1 | 1 | N/A | 1 | 1 | − | 1 | 1 | 1 |  |  |  |  |  |  |  |  |  |  |  |  |  |  |
| Boo (2005) | 1 | 1 | 1 | 1 | 1 | 1 | 1 | 1 | − | − | − | 1 | 1 | 1 | 1 | N/A | 1 | 1 | 1 | 1 | N/A | 1 | 1 |  | 1 | 1 | 1 | N/A | 1 | 1 | − | 1 | 1 | − |  |  |  |  |  |  |  |  |  |  |  |  |  |  |
| Calbo, 2011 | − | 1 | 1 | 1 | 1 | 1 | 1 | 1 | − | 1 | − | 1 | 1 | 1 | 1 | N/A | 1 | 1 | 1 | 1 | N/A | − | 1 |  | 1 | 1 | − | N/A | 1 | 1 | − | 1 | 1 | 1 |  |  |  |  |  |  |  |  |  |  |  |  |  |  |
| Cassettari (2009) | − | 1 | 1 | 1 | 1 | 1 | 1 |  | − | − | − | 1 | 1 | 1 | 1 | N/A | 1 | 1 | 1 | 1 | N/A | − | 1 |  | 1 | 1 | 1 | N/A | 1 | 1 | 1 | 1 | 1 | 1 |  |  |  |  |  |  |  |  |  |  |  |  |  |  |
| Demir (2008) | 1 | 1 | 1 | 1 | 1 | 1 | 1 | 1 | − | 1 | − | 1 | 1 | 1 | 1 | N/A | 1 | 1 | 1 | 1 | N/A | − | 1 |  | 1 | 1 | 1 | N/A | 1 | 1 | − | 1 | 1 | 1 |  |  |  |  |  |  |  |  |  |  |  |  |  |  |
| Demirdag (2010) | 1 | 1 | 1 | 1 | 1 | 1 | 1 | 1 | − | 1 | − | 1 | 1 | 1 | 1 | N/A | 1 | 1 | 1 | 1 | N/A | − | 1 |  | 1 | 1 | 1 | N/A | 1 | 1 | − | 1 | 1 | 1 |  |  |  |  |  |  |  |  |  |  |  |  |  |  |
| Gupta (2004) | − | 1 | 1 | 1 | 1 | 1 | 1 | 1 | − | 1 | − | 1 | 1 | 1 | 1 | N/A | 1 | 1 | 1 | 1 | N/A | − | 1 |  | 1 | 1 | 1 | N/A | 1 | 1 | 1 | 1 | − | − |  |  |  |  |  |  |  |  |  |  |  |  |  |  |
| Kang (2004)³ | − | 1 | 1 | 1 | 1 | 1 | 1 | 1 | − | 1 | − | 1 | 1 | 1 | 1 | N/A | 1 | 1 | 1 | 1 | N/A | − | 1 |  | 1 | 1 | 1 | N/A | 1 | 1 | 1 | 1 | − | − |  |  |  |  |  |  |  |  |  |  |  |  |  |  |
| Kuo (2007) | 1 | 1 | 1 | 1 | 1 | 1 | 1 | 1 | − | 1 | − | 1 | 1 | 1 | 1 | N/A | 1 | 1 | 1 | 1 | N/A | − | 1 |  | 1 | 1 | 1 | N/A | 1 | 1 | − | 1 | 1 | − |  |  |  |  |  |  |  |  |  |  |  |  |  |  |
| Lee (2004) | − | 1 | 1 | 1 | 1 | 1 | 1 | 1 | 1 | 1 | − | 1 | 1 | 1 | 1 | N/A | 1 | 1 | 1 | 1 | N/A | 1 | 1 |  | 1 | 1 | 1 | N/A | 1 | 1 | − | 1 | 1 | − |  |  |  |  |  |  |  |  |  |  |  |  |  |  |
| Lee (2013) | − | 1 | 1 | 1 | − | 1 | 1 |  | − | 1 | − | 1 | 1 | 1 | 1 | N/A | 1 | 1 | 1 | − | N/A | − | 1 |  | 1 | 1 | 1 | N/A | 1 | 1 | 1 | 1 | 1 | 1 |  |  |  |  |  |  |  |  |  |  |  |  |  |  |
| Li (2013)³ | − | 1 | 1 | 1 | − | 1 | 1 |  | − | 1 | − | 1 | 1 | 1 | 1 | N/A | 1 | 1 | 1 | 1 | N/A | − | 1 |  | 1 | 1 | 1 | N/A | 1 | 1 | − | 1 | 1 | 1 |  |  |  |  |  |  |  |  |  |  |  |  |  |  |
| Lytsy (2010) | 1 | 1 | 1 | 1 | 1 | 1 | 1 | 1 | − | 1 | 1 | 1 | 1 | 1 | 1 | N/A | 1 | 1 | 1 | 1 | N/A | 1 | 1 |  | 1 | 1 | 1 | N/A | 1 | 1 | − | 1 | 1 | 1 |  |  |  |  |  |  |  |  |  |  |  |  |  |  |
| Martins (2006) | − | 1 | 1 | 1 | 1 | 1 | 1 | − | − | 1 | − | 1 | 1 | 1 | 1 | 1 | 1 | 1 | 1 | 1 | N/A | − | 1 | − | 1 | − | 1 | N/A | 1 | 1 | − | 1 | 1 | 1 |  |  |  |  |  |  |  |  |  |  |  |  |  |  |
| Mosqueda-Gomez (2008)³ | 1 | 1 | 1 | 1 | 1 | 1 | 1 | − | − | 1 | − | 1 | 1 | 1 | 1 | N/A | 1 | 1 | 1 | 1 | N/A | − | 1 |  | 1 | − | 1 | N/A | 1 | 1 | − | 1 | 1 | 1 |  |  |  |  |  |  |  |  |  |  |  |  |  |  |
| Pena (1997) | − | 1 | 1 | 1 | − | 1 | 1 |  | − | 1 | − | 1 | 1 | 1 | 1 | N/A | 1 | 1 | 1 | 1 | N/A | − | 1 |  | 1 | 1 | 1 | N/A | 1 | 1 | − | 1 | 1 | 1 |  |  |  |  |  |  |  |  |  |  |  |  |  |  |
| Rettedal (2013) | − | 1 | 1 | 1 | − | 1 | − | − | − | 1 | − | 1 | 1 | 1 | 1 | N/A | 1 | 1 | 1 | 1 | N/A | − | 1 | − | 1 | 1 | 1 | N/A | 1 | 1 | − | 1 | 1 | 1 |  |  |  |  |  |  |  |  |  |  |  |  |  |  |
| Saely (2011) | − | 1 | 1 | 1 | 1 | 1 | 1 | 1 | − | 1 | − | 1 | 1 | 1 | 1 | N/A | 1 | 1 | 1 | 1 | N/A | − | 1 |  | 1 | − | 1 | N/A | 1 | 1 | 1 | 1 | 1 | 1 |  |  |  |  |  |  |  |  |  |  |  |  |  |  |
| Sanchez (2012) | 1 | 1 | 1 | 1 | 1 | 1 | 1 | 1 | − | 1 | − | 1 | 1 | 1 | 1 | N/A | 1 | 1 | 1 | − | N/A | 1 | 1 |  | 1 | 1 | 1 | N/A | 1 | 1 | 1 | 1 | 1 | − |  |  |  |  |  |  |  |  |  |  |  |  |  |  |
| Tumbarello (2006) | − | 1 | 1 | 1 | 1 | 1 | 1 | 1 | − | 1 | − | 1 | 1 | 1 | 1 | N/A | 1 | 1 | 1 | 1 | N/A | − | 1 |  | 1 | 1 | 1 | N/A | 1 | 1 | − | 1 | 1 | 1 |  |  |  |  |  |  |  |  |  |  |  |  |  |  |
| Wener (2010) | − | 1 | 1 | 1 | 1 | 1 | 1 | 1 | − | 1 | − | 1 | 1 | 1 | 1 | N/A | 1 | 1 | 1 | 1 | N/A | 1 | 1 |  | 1 | 1 | 1 | N/A | 1 | 1 | 1 | 1 | 1 | − |  |  |  |  |  |  |  |  |  |  |  |  |  |  |

Abbreviations: NA, not available

| **S3 Table C1. Quality assessment scores of the 15 case-control studies that were included in the random-effects meta-analysis study, using the Newcastle Ottawa Scale.** | | | | | | | | | | | | |
| --- | --- | --- | --- | --- | --- | --- | --- | --- | --- | --- | --- | --- |
| **Study** | **Selection** | | | | **Comparability** | | **Exposure** | | | |  |  |
|  | 1 | 2 | 3 | 4 | 1a | 1b | 1a | 1b | 2 | 3 |  |  |
| Asensio (2000) | − | * | − | * | * | * | * | − | * | − |  |  |
| Boo (2005) | − | * | − | * | * | * | − | − | * | − |  |  |
| Calbo (2011) | − | * | − | * | * | * | * | − | * | − |  |  |
| Demir (2008) | − | * | − | * | * | * | * | − | * | − |  |  |
| Demirdag (2010) | − | * | − | * | * | * | * | − | * | − |  |  |
| Gupta (2004) | − | * | − | * | * | * | * | − | * | − |  |  |
| Kang (2004) | − | * | − | * | * | * | * | − | * | − |  |  |
| Kuo (2007) | − | * | − | * | * | * | * | − | * | − |  |  |
| Lee (2004) | − | * | − | * | * | * | * | − | * | − |  |  |
| Mosqueda-Gomez (2008) | − | * | − | * | * | * | * | − | * | − |  |  |
| Saely (2011) | − | * | − | * | * | * | * | − | * | − |  |  |
| Sanchez (2012) | − | * | − | * | * | * | * | − | * | − |  |  |
| Tumbarello (2006) | − | * | − | * | * | * | * | − | * | − |  |  |
| Lytsy (2010) | − | * | − | * | * | * | * | − | * | − |  |  |
| Wener (2010) | − | * | − | * | * | * | * | − | * | − |  |  |

**S3 Table C2. Quality assessment scores of the three cohort studies that were included in the random-effects meta-analysis study, using the Newcastle Ottawa Scale.**

| **Study** | **Selection** | | | |  |  | **Comparability** | | **Outcome** | | | |  |
| --- | --- | --- | --- | --- | --- | --- | --- | --- | --- | --- | --- | --- | --- |
|  | 1a | 1b | 2 | 3a | 3b | 4 | 1a | 1b | 1a | 1b | 2 | 3a | 3b |
| Abdel-Hady (2008) | − | − | − | * | − | * | * | * | − | * | * | * | − |
| Martins (2006) | − | − | − | − | − | * | * | * | − | − | * | − | * |
| Rettedal (2013) | − | − | − | * | − | * | * | * | − | − | − | − | * |

| **S3 Table D. Quality assessment scores of 120 articles that were included in the cluster analysis study.** | | | | | | | | | | | | | | | | | | | | | | | | | | | |
| --- | --- | --- | --- | --- | --- | --- | --- | --- | --- | --- | --- | --- | --- | --- | --- | --- | --- | --- | --- | --- | --- | --- | --- | --- | --- | --- | --- |
| **Study^a^** | **STROME-ID Items** | | | | | | | | | | | | | | | | | | | | | | | | | | **STROME ID Score^b^: (n/max)** |
|  | **1** | **2** | **3** | | **4** | | | | | | **5** | | **6** | | **8** | **9** | **10** | **12** | | | **13** | | **14** | **16** | **19** | **23** |  |
|  |  |  |  | | **1** | | **2** | **3** | **4** | |  | |  | |  |  |  | **1** | **2** | | **1** | **2** |  |  |  |  |  |
| Abecasis (2011) | − | 1 | − | | 1 | | 1 | 1 | 0.5 | | 1 | | 1 | | 1 | − | − | NA | − | | 1 | 1 | 1 | − | 1 | 1 | 12.5/19 |
| Ahmed (2012) | − | 1 | 0.5 | | − | | 1 | − | 0.5 | | 0.5 | | 1 | | 1 | − | − | NA | − | | 1 | 1 | 1 | − | 1 | − | 9.5/19 |
| Akpaka (2010) | 1 | 1 | 1 | | − | | 1 | − | 0.5 | | 0.5 | | 1 | | 1 | − | − | NA | − | | 1 | 1 | 1 | − | 1 | − | 11/19 |
| Al Sweih (2011) | − | 1 | 0.5 | | − | | 1 | 1 | 0.5 | | 0.5 | | 1 | | 1 | − | − | NA | − | | 1 | 1 | 1 | 1 | 1 | − | 11.5/19 |
| Alcantar-Curiel (2004) | − | 1 | 1 | | − | | 1 | 1 | 0.5 | | 1 | | 1 | | 1 | − | − | NA | 1 | | 1 | 1 | 1 | − | 1 | − | 12.5/19 |
| Anderson (2006) | − | 1 | − | | − | | 1 | 1 | 0.5 | | 1 | | 1 | | − | − | − | NA | − | | 1 | 1 | 1 | − | − | − | 8.5/19 |
| Andrade (2004) | − | 1 | 1 | | − | | 1 | − | 0.5 | | 0.5 | | 1 | | 1 | − | − | NA | − | | 1 | 1 | 1 | − | − | − | 9/19 |
| Apisarnthanarak (2008) | 1 | 1 | 1 | | 1 | | 1 | 1 | 0.5 | | 1 | | 1 | | 1 | − | − | NA | − | | 1 | 1 | 1 | 1 | 1 | − | 14.5/19 |
| Arpin (2000) | − | 1 | 1 | | − | | 1 | 1 | 0.5 | | 1 | | 1 | | 1 | − | − | NA | − | | 1 | 1 | 1 | − | 1 | − | 11.5/19 |
| Asensio (2000) | − | 1 | 0.5 | | − | | 1 | 1 | 0.5 | | 1 | | 1 | | − | − | − | NA | − | | 1 | 1 | − | − | − | − | 8/19 |
| Bagattini (2006) | 1 | 1 | 1 | | − | | 1 | − | 0.5 | | 0.5 | | 1 | | 1 | − | − | NA | − | | 1 | 1 | 1 | − | 1 | − | 11/19 |
| Barroso (2000) | − | 1 | 0.5 | | − | | 1 | − | 0.5 | | 0.5 | | 1 | | 1 | − | − | NA | − | | 1 | 1 | 1 | − | 1 | − | 9.5/19 |
| Benenson (2013) | − | 0.5 | − | | − | | 1 | 1 | 0.5 | | 1 | | 1 | | − | − | − | NA | − | | 1 | 1 | − | − | 1 | 1 | 9/19 |
| Ben-Hamouda (2004) | − | 1 | 1 | | − | | 1 | − | 0.5 | | 0.5 | | 1 | | − | − | − | NA | − | | 1 | 1 | 1 | − | 1 | − | 9/19 |
| Bennett (2010) | − | 1 | 1 | | − | | 1 | 1 | 0.5 | | 1 | | 1 | | 1 | − | − | NA | − | | 1 | 1 | 1 | − | 1 | 1 | 12.5/19 |
| Bermudes (1997) | 1 | 1 | 1 | | − | | 1 | − | 0.5 | | 0.5 | | 1 | | 1 | − | − | NA | − | | 1 | 1 | 1 | − | 1 | − | 11/19 |
| Bingen (1993) | 1 | 1 | 0.5 | | 0.5 | | 1 | − | 0.5 | | − | | 1 | | 1 | − | − | NA | − | | 1 | 1 | 1 | − | 1 | − | 10.5/19 |
| Boszczowski (2005) | − | 1 | − | | − | | 1 | − | 0.5 | | 1 | | 1 | | − | − | − | NA | − | | 1 | 1 | − | − | 1 | − | 7.5/19 |
| Bouallegue-Godet (2005) | − | 1 | 1 | | − | | 1 | − | 0.5 | | 1 | | 1 | | − | − | − | NA | − | | 1 | 1 | − | 1 | 1 | − | 9.5/19 |
| Boukadida (2002) | − | 0.5 | 1 | | − | | 0.5 | − | 0.5 | | 1 | | 1 | | − | − | − | NA | − | | 1 | 1 | − | − | 1 | − | 7.5/19 |
| Branger (1998) | − | 1 | 1 | | − | | 1 | − | 0.5 | | 1 | | 1 | | 1 | − | − | NA | − | | 1 | 1 | 1 | 1 | 1 | − | 11.5/19 |
| Brinas (2004) | − | 1 | 1 | | − | | 1 | − | 0.5 | | 1 | | 1 | | 1 | − | − | NA | − | | 1 | 1 | 1 | − | 1 | − | 10.5/19 |
| Calbo (2011) | − | 1 | 1 | | − | | 1 | 1 | 0.5 | | 1 | | 1 | | 1 | − | − | NA | − | | 1 | 1 | 1 | − | 1 | − | 11.5/19 |
| Cantey (2013) | − | 1 | − | | − | | 1 | 1 | 0.5 | | 1 | | 1 | | 1 | − | − | NA | − | | 1 | 1 | 1 | − | 1 | − | 10.5/19 |
| Carpentier (2012) | − | 1 | 0.5 | | − | | 1 | 1 | 0.5 | | 1 | | 1 | | − | − | − | NA | − | | 1 | 1 | 1 | 1 | 1 | − | 11/19 |
| Carrer (2009) | − | 1 | 1 | | − | | 1 | 1 | 0.5 | | 1 | | 1 | | 1 | − | − | NA | − | | 1 | 1 | 1 | 1 | 1 | − | 12.5/19 |
| Cassettari (2009) | − | 1 | 1 | | − | | 1 | − | 0.5 | | 1 | | 1 | | − | − | − | NA | − | | 1 | 1 | 1 | 1 | 1 | − | 10.5/19 |
| Chanawong (2001) | − | 1 | 1 | | − | | 1 | − | 0.5 | | 0.5 | | 1 | | 1 | − | − | NA | − | | 1 | 1 | 1 | − | 1 | − | 10/19 |
| Christian (2010) | 1 | 1 | 1 | | − | | 1 | − | 0.5 | | 0.5 | | 1 | | − | − | − | NA | − | | 1 | 1 | − | − | 1 | 1 | 10/19 |
| Conte (2005) | − | 1 | 1 | | − | | 1 | − | 0.5 | | 1 | | 1 | | − | − | − | NA | − | | 1 | 1 | − | 1 | 1 | − | 9.5/19 |
| Dahmen (2010) | 1 | 1 | 1 | | − | | 1 | − | 0.5 | | 0.5 | | 1 | | 1 | − | − | NA | − | | 1 | 1 | 1 | − | 1 | − | 11/19 |
| Dashti (2010) | − | 1 | − | | − | | 1 | − | 0.5 | | 0.5 | | 1 | | 1 | − | − | NA | − | | 1 | 1 | 1 | − | 1 | − | 9/19 |
| de Almeida (2005) | − | 1 | 1 | | 1 | | 1 | 1 | 0.5 | | 1 | | 1 | | − | − | − | NA | − | | 1 | 1 | 1 | − | 1 | 1 | 12.5/19 |
| de Oliveira Garcia (2008) | − | 1 | 1 | | − | | 1 | − | 0.5 | | 0.5 | | 1 | | 1 | − | − | NA | − | | 1 | 1 | 1 | − | 1 | − | 10/19 |
| Decre (1998) | − | 1 | 1 | | 1 | | 1 | 1 | 0.5 | | 1 | | 1 | | 1 | − | − | NA | − | | 1 | 1 | 1 | 1 | 1 | − | 13.5/19 |
| Dedeic-Ljubovic (2010) | − | 1 | 1 | | − | | 1 | 1 | 0.5 | | 0.5 | | 0.5 | | 0.5 | − | − | NA | − | | 1 | 1 | 1 | 1 | 1 | − | 11/19 |
| Demir (2008) | − | 1 | − | | − | | 1 | 1 | 0.5 | | 1 | | 1 | | − | − | − | NA | − | | 1 | 1 | − | 1 | 1 | 1 | 10.5/19 |
| Demirdag (2010) | 1 | 1 | − | | − | | 1 | 1 | 0.5 | | 1 | | 1 | | − | − | − | NA | − | | 1 | 1 | − | − | 1 | − | 9.5/19 |
| Do Carmo Filho (2008) | − | 1 | 1 | | − | | 1 | − | 0.5 | | 0.5 | | 1 | | − | − | − | NA | − | | 1 | 1 | 1 | − | 1 | − | 9/19 |
| Dolejska (2012) | − | 1 | 1 | | − | | 1 | 1 | 0.5 | | 1 | | 1 | | 1 | − | − | NA | − | | 1 | 1 | 1 | 1 | 1 | 1 | 13.5/19 |
| Dumpis (2010) | − | 1 | 1 | | − | | 1 | 1 | 0.5 | | 0.5 | | 1 | | − | − | − | NA | − | | 1 | 1 | 1 | 1 | 1 | − | 11/19 |
| Elhani (2006) | − | 1 | 1 | | 0.5 | | 1 | − | 0.5 | | 0.5 | | 1 | | − | − | − | NA | − | | 1 | 1 | − | − | 1 | − | 8.5/19 |
| Elhani (2010) | 1 | 1 | 0.5 | | − | | 1 | − | 0.5 | | 0.5 | | 1 | | 1 | − | − | NA | − | | 1 | 1 | 1 | − | 1 | − | 10.5/19 |
| Fiett (2000) | − | 1 | 1 | | − | | 1 | − | 0.5 | | 0.5 | | 1 | | 1 | − | − | NA | − | | 1 | 1 | 1 | − | 1 | − | 10/19 |
| Filozov (2009) | − | 1 | 0.5 | | 0.5 | | 1 | 1 | 0.5 | | 1 | | 1 | | − | − | − | NA | − | | 1 | 1 | − | − | 1 | − | 9.5/19 |
| French (1996) | − | 1 | 0.5 | | − | | 1 | − | 0.5 | | − | | 1 | | 1 | − | − | NA | − | | 1 | 1 | 1 | − | 1 | − | 9/19 |
| Gaillot (1998) | − | 1 | 1 | | − | | 1 | − | 0.5 | | 0.5 | | 1 | | 1 | − | − | NA | − | | 1 | 1 | 1 | − | 1 | − | 10/19 |
| Garza-Gonzalez (2011) | − | 1 | 1 | | 1 | | 1 | − | 0.5 | | 0.5 | | 1 | | 1 | − | − | NA | − | | 1 | 1 | 1 | 1 | 1 | − | 12/19 |
| Giraud-Morin (2003) | − | 1 | 1 | | 0.5 | | 1 | − | 0.5 | | 0.5 | | 1 | | 1 | − | − | NA | − | | 1 | 1 | 1 | − | 1 | − | 10.5/19 |
| Gonzalez (2011) | − | 1 | 1 | | − | | 1 | 1 | 0.5 | | 1 | | 1 | | 1 | − | − | NA | − | | 1 | 1 | 1 | − | 1 | 1 | 12.5/19 |
| Gonzalez-Vertiz (2001) | − | 1 | 1 | | − | | 1 | 1 | 0.5 | | 1 | | 1 | | 1 | − | − | NA | − | | 1 | 1 | − | − | 1 | − | 10.5/19 |
| Gray (2012) | − | 1 | 0.5 | | − | | 1 | 1 | 0.5 | | 1 | | 1 | | − | − | − | NA | − | | 1 | 1 | − | − | 1 | 1 | 10/19 |
| Gruteke (2003) | − | 1 | − | | − | | 1 | 1 | 0.5 | | 1 | | 1 | | 1 | − | − | NA | − | | 1 | 1 | 1 | − | 1 | − | 10.5/19 |
| Gundes (2005) | − | 1 | 0.5 | | − | | 1 | − | 0.5 | | 0.5 | | 1 | | 1 | − | − | NA | − | | 1 | 1 | 1 | − | − | − | 8.5/19 |
| Gupta (2004) | − | 1 | − | | − | | 1 | 1 | 0.5 | | 1 | | 1 | | − | − | − | NA | − | | 1 | 1 | − | − | 1 | 1 | 9.5/19 |
| Guyot (2012) | − | 1 | 1 | | − | | 1 | 1 | 0.5 | | 1 | | 1 | | 1 | − | − | NA | − | | 1 | 1 | 1 | 1 | 1 | − | 12.5/19 |
| Harris (2007) | − | 1 | 1 | | 0.5 | | 1 | 1 | 0.5 | | 1 | | 1 | | − | − | − | NA | − | | 1 | 1 | − | 1 | 1 | − | 11/19 |
| Hirakata (1999) | − | 1 | NA | | − | | 1 | NA | 0.5 | | 1 | | 1 | | 1 | − | NA | NA | NA | | 1 | 1 | 1 | 1 | 1 | − | 10.5/15^c^ |
| Hollander (2001) | − | 1 | 0.5 | | − | | 1 | − | − | | − | | 1 | | − | − | − | NA | − | | 1 | 1 | − | − | 1 | − | 6.5/19 |
| Hosbul (2011) | − | 1 | 1 | | − | | 1 | − | − | | 1 | | 1 | | − | − | − | NA | − | | 1 | 1 | − | − | 1 | − | 8/19 |
| Jeong (2001) | − | 0.5 | 1 | | − | | 1 | − | 0.5 | | − | | 1 | | 1 | − | − | NA | − | | 1 | 1 | 1 | − | 1 | − | 9/19 |
| Jutersek (2003) | − | 1 | 1 | | − | | 1 | − | 0.5 | | 0.5 | | 1 | | 1 | − | − | NA | − | | 1 | 1 | 1 | − | 1 | − | 10/19 |
| Kac (2004) | 1 | 1 | 1 | | − | | 1 | − | 0.5 | | 0.5 | | 1 | | 1 | − | − | NA | − | | 1 | 1 | − | 1 | 1 | − | 11/19 |
| Komatsu (2001) | − | 1 | 1 | | − | | 1 | − | 0.5 | | 1 | | 1 | | 1 | − | − | NA | − | | 1 | 1 | 1 | 1 | 1 | − | 11.5/19 |
| Kristof (2007) | 1 | 1 | 1 | | − | | 1 | 1 | 0.5 | | 1 | | 1 | | 1 | − | − | NA | − | | 1 | 1 | 1 | − | 1 | − | 12.5/19 |
| Langer (2009) | − | 1 | 0.5 | | − | | 1 | 1 | 0.5 | | 1 | | 1 | | − | − | − | NA | − | | 1 | 1 | − | − | 1 | − | 9/19 |
| Laurent (2008) | − | 1 | − | | − | | 1 | 1 | 0.5 | | 1 | | 1 | | 1 | − | − | NA | − | | 1 | 1 | 1 | − | 1 | − | 10.5/19 |
| Lavigne (2004) | 1 | 1 | 1 | | 0.5 | | 1 | 1 | 0.5 | | 1 | | 1 | | 1 | − | − | NA | − | | 1 | 1 | 1 | 1 | 1 | − | 14/19 |
| Lin (2010) | 1 | 1 | 1 | | 0.5 | | 1 | − | 0.5 | | 0.5 | | 1 | | 1 | − | − | NA | − | | 1 | 1 | 1 | 1 | 1 | − | 12.5/19 |
| Liu (1998) | 1 | 1 | 1 | | 0.5 | | 1 | − | 0.5 | | 1 | | 1 | | 1 | − | − | NA | − | | 1 | 1 | 1 | 1 | 1 | − | 13/19 |
| Lowe (2012) | − | 1 | − | | − | | 1 | 1 | − | | 0.5 | | 1 | | − | − | − | NA | − | | 1 | 1 | − | − | 1 | − | 7.5/19 |
| Lucet (1996) | − | 1 | 0.5 | | − | | 1 | 1 | 0.5 | | 1 | | 1 | | − | − | − | NA | − | | 1 | 1 | − | − | 1 | − | 9/19 |
| Lytsy ( 2008 | − | 1 | 1 | | 0.5 | | 1 | − | 0.5 | | 1 | | 1 | | 1 | − | − | NA | − | | 1 | 1 | 1 | − | − | − | 10/19 |
| Macrae (2001) | − | 1 | 0.5 | | − | | 1 | − | 0.5 | | − | | 1 | | 1 | − | − | NA | − | | 1 | 1 | 1 | − | 1 | − | 9/19 |
| Manageiro (2012) | − | 1 | 0.5 | | − | | 1 | − | 0.5 | | 0.5 | | 1 | | 1 | − | − | NA | − | | 1 | 1 | 1 | 1 | 1 | − | 10.5/19 |
| Mangeney (2000) | − | 1 | 1 | | 0.5 | | 1 | 1 | 0.5 | | 1 | | 1 | | 1 | − | − | NA | − | | 1 | 1 | 1 | 1 | 1 | − | 13/19 |
| Mantilla (2006) | − | 1 | 1 | | − | | 1 | − | 0.5 | | 1 | | 1 | | 1 | − | − | NA | − | | 1 | 1 | 1 | 1 | 1 | − | 11.5/19 |
| Marcade (2013) | 1 | 1 | 1 | | − | | 0.5 | 1 | 0.5 | | 1 | | 1 | | 1 | − | − | NA | − | | 1 | 1 | 1 | − | 1 | − | 12/19 |
| Marchese (1996) | − | 1 | 0.5 | | − | | 1 | − | − | | 0.5 | | 0.5 | | 1 | − | − | NA | − | | 1 | 1 | 1 | − | 1 | − | 8.5/19 |
| Martínez-Aguilar (2001) | − | − | 1 | | − | | 1 | 1 | 0.5 | | 1 | | 1 | | − | − | − | NA | − | | 1 | 1 | 1 | − | 1 | − | 9.5/19 |
| Martins (2006) | − | 1 | − | | 0.5 | | 1 | 1 | 0.5 | | 1 | | 1 | | − | − | − | NA | − | | 1 | 1 | − | 1 | 1 | − | 10/19 |
| Martins-Loureiro (2001) | 1 | 1 | 1 | | 0.5 | | 1 | 1 | 0.5 | | 1 | | 1 | | − | − | − | NA | − | | 1 | 1 | − | 1 | 1 | − | 12/19 |
| Mena (2006) | − | 1 | 0.5 | | − | | 1 | − | 0.5 | | 1 | | 1 | | 1 | − | − | NA | − | | 1 | 1 | 1 | − | − | − | 9/19 |
| Minarini (2008) | − | 1 | 1 | | − | | 1 | − | 0.5 | | 0.5 | | 1 | | 1 | − | − | NA | − | | 1 | 1 | 1 | − | 1 | − | 10/19 |
| Mosqueda-Gomez (2008) | 1 | 1 | 1 | | 0.5 | | 1 | 1 | 0.5 | | 1 | | 1 | | 1 | − | − | NA | − | | 1 | 1 | 1 | − | 1 | 1 | 14/19 |
| Moustaoui (2004) | − | 1 | 1 | | − | | 1 | − | 0.5 | | − | | 0.5 | | 1 | − | − | NA | − | | 1 | 1 | − | − | 1 | − | 8/19 |
| Mshana (2013) | 1 | 1 | 1 | | 0.5 | | 1 | 1 | 0.5 | | 1 | | 1 | | 1 | − | − | NA | − | | 1 | 1 | 1 | 1 | 1 | − | 14/19 |
| Muro (2012) | 1 | 1 | 0.5 | | 1 | | 1 | 1 | 0.5 | | 1 | | 1 | | 1 | − | − | NA | − | | 1 | 1 | 1 | 1 | 1 | − | 14/19 |
| Nedjai (2012) | − | 1 | 1 | | 0.5 | | 1 | − | 0.5 | | 1 | | 1 | | 1 | − | − | NA | − | | 1 | 1 | 1 | 1 | 1 | − | 12/19 |
| Nouvellon (1994) | − | 1 | 1 | | − | | 1 | − | 0.5 | | 1 | | 1 | | 1 | − | − | NA | − | | 1 | 1 | 1 | − | 1 | − | 10.5/19 |
| Paniara (2000) | − | 1 | 1 | | − | | 1 | − | − | | 0.5 | | 1 | | 1 | − | − | NA | − | | 1 | 1 | 1 | − | 1 | − | 9.5/19 |
| Parasakthi (2000) | 1 | 1 | 1 | | 0.5 | | 1 | − | 0.5 | | 1 | | 1 | | − | − | − | NA | − | | 1 | 1 | − | 1 | 1 | − | 11/19 |
| Pena (1998) | 1 | 1 | 1 | | 1 | | 1 | 1 | 0.5 | | 1 | | 1 | | − | − | − | NA | − | | 1 | 1 | − | − | 1 | − | 11.5/19 |
| Pessoa-Silva (2003) | − | 1 | − | | − | | 1 | 1 | 0.5 | | 1 | | 1 | | 1 | − | − | NA | − | | 1 | 1 | − | − | 1 | 1 | 10.5/19 |
| Rebuck (2000) | − | 1 | 1 | | 1 | | 1 | 1 | 0.5 | | 1 | | 1 | | − | − | − | NA | − | | 1 | 1 | − | − | 1 | − | 10.5/19 |
| Rettedal (2012) | − | 1 | 0.5 | | 1 | | 1 | 1 | 0.5 | | 1 | | 1 | | 1 | − | − | NA | − | | 1 | 1 | 1 | − | 1 | − | 12/19 |
| Rice (1996) | 1 | 1 | 1 | | 1 | | 1 | − | 0.5 | | 1 | | 1 | | 1 | − | − | NA | − | | 1 | − | 1 | − | 1 | − | 11.5/19 |
| Roy (2013) | 1 | 1 | 1 | | − | | 1 | − | 0.5 | | 1 | | 1 | | 1 | − | − | NA | − | | 1 | 1 | 1 | − | 1 | − | 11.5/19 |
| Royle (1999) | − | 1 | − | | − | | 1 | 1 | − | | 1 | | 1 | | − | − | − | NA | − | | 1 | 1 | − | − | 1 | − | 8/19 |
| Sanchez (2012) | − | 1 | − | | 0.5 | | 1 | 1 | − | | 1 | | 1 | | − | − | − | NA | − | | 1 | 1 | − | − | 1 | − | 8.5/19 |
| Severin (2010) | 1 | 1 | 1 | | 0.5 | | 1 | − | 0.5 | | 0.5 | | 1 | | 1 | − | − | NA | − | | 1 | 1 | 1 | 1 | 1 | − | 12.5/19 |
| Shannon (1998) | − | 1 | 0.5 | | − | | 1 | − | 0.5 | | 1 | | 1 | | 1 | − | − | NA | − | | 1 | 1 | 1 | − | 1 | − | 10/19 |
| Shu (2010) | − | 1 | 1 | | 0.5 | | 1 | 1 | 0.5 | | 1 | | 1 | | 1 | − | − | NA | − | | 1 | 1 | 1 | − | 1 | − | 12/19 |
| Silva (2001) | − | 1 | 0.5 | | − | | 1 | − | 0.5 | | 0.5 | | 1 | | 1 | − | − | NA | − | | 1 | 1 | 1 | − | 1 | − | 9.5/19 |
| Somily (2014) | − | 1 | 1 | | 1 | | 1 | 1 | 0.5 | | 1 | | 1 | | − | − | − | NA | − | | − | − | − | − | 1 | − | 8.5/19 |
| Starlander (2012) | − | − | NA | | − | | 1 | NA | 0.5 | | 1 | | 1 | | − | − | NA | NA | NA | | 1 | 1 | − | − | 1 | − | 6.5/15^c^ |
| Sumer (2013) | − | 1 | − | | 0.5 | | 1 | − | 0.5 | | 1 | | 1 | | − | − | − | NA | − | | 1 | 1 | − | 1 | 1 | 1 | 10/19 |
| Szabo (1999) | 1 | 1 | − | | 0.5 | | 1 | 1 | 0.5 | | 1 | | 1 | | 1 | − | − | NA | − | | 1 | 1 | − | − | 1 | − | 11/19 |
| Tamma (2012) | − | 1 | − | | − | | 1 | 1 | − | | 1 | | 1 | | − | − | − | NA | − | | 1 | 1 | 1 | − | 1 | − | 9/19 |
| Tollentino (2011) | − | 1 | 0.5 | | 0.5 | | 1 | − | 0.5 | | 0.5 | | 1 | | 1 | − | − | NA | − | | 1 | 1 | 1 | 1 | 1 | − | 11/19 |
| Tumbarello (2006) | 1 | 1 | 1 | | − | | 1 | 1 | 0.5 | | 1 | | 1 | | 1 | − | − | NA | − | | 1 | 1 | 1 | − | 1 | − | 12.5/19 |
| Valverde (2008) | 1 | 1 | 1 | | − | | 1 | − | 0.5 | | 0.5 | | 1 | | 1 | − | − | NA | − | | 1 | 1 | 1 | − | 1 | − | 11/19 |
| van 't Veen (2005) | − | − | 1 | | 0.5 | | 1 | 1 | 0.5 | | 1 | | 1 | | − | − | − | NA | − | | 1 | 1 | − | 1 | 1 | − | 10/19 |
| Velasco (2009) | − | 1 | 1 | | − | | 1 | − | 0.5 | | 0.5 | | 1 | | 1 | − | − | NA | − | | 1 | 1 | 1 | − | 1 | − | 10/19 |
| Venezia (1995) | 1 | 1 | 1 | | − | | 1 | − | 0.5 | | 1 | | 1 | | 1 | − | − | NA | − | | 1 | 1 | 1 | − | 1 | − | 11.5/19 |
| Villari (1998) | 1 | − | 1 | | − | | 1 | 1 | − | | 1 | | 1 | | 1 | − | − | NA | − | | 1 | 1 | − | − | 1 | − | 10/19 |
| Wollheim (2011) | − | 1 | 1 | | − | | 1 | 1 | 0.5 | | 1 | | 1 | | 1 | − | − | NA | − | | 1 | 1 | 1 | − | 1 | − | 11.5/19 |
| Wu (2006) | − | 1 | 1 | | 0.5 | | 1 | − | 0.5 | | 0.5 | | 1 | | 1 | − | − | NA | 1 | | 1 | 1 | 1 | − | 1 | − | 11.5/19 |
| Yu (2009) | − | 1 | − | | − | | 1 | − | 0.5 | | 0.5 | | 1 | | 1 | − | − | NA | − | | 1 | 1 | 1 | − | 1 | − | 9/19 |
| Zhang (2012) | − | 1 | 0.5 | | 0.5 | | 1 | − | 0.5 | | 1 | | 1 | | 1 | − | − | NA | − | | 1 | 1 | 1 | 1 | 1 | 1 | 12.5/19 |
| Abbreviations: STROME-ID, Strengthening the reporting of molecular epidemiology for infectious disease. NA, not available because the coding was not suitable. | | | | | | | | | | | | | | | | | | | | | | | | | | | |
| ^a^ Studies are reported by first author and year. | | | | | | |  |  |  | | |  | |  |  |  |  |  | |  |  |  |  |  |  |  |  |
| ^b^ n, study score; maximum score: 19 except for case report. | | | | | | | | | | | |  |  | |  |  |  |  | |  |  |  |  |  |  |  |  |
| ^c^ case report, maximum score: 15. | | |  |  | |  |  |  | |  | |  |  | |  |  |  |  | |  |  |  |  |  |  |  |  |
|  | | | | | | | | | |  | |  |  | |  |  |  |  | |  |  |  |  |  |  |  |  |
